# Spent Hop (*Humulus lupulus* L.) Extract and Its Flaxseed Polysaccharide-Based Encapsulates Attenuate Inflammatory Bowel Diseases Through the Nuclear Factor-Kappa B, Extracellular Signal-Regulated Kinase, and Protein Kinase B Signalling Pathways

**DOI:** 10.3390/cells14141099

**Published:** 2025-07-17

**Authors:** Miłosz Caban, Katarzyna Owczarek, Justyna Rosicka-Kaczmarek, Karolina Miśkiewicz, Joanna Oracz, Wojciech Pawłowski, Karolina Niewinna, Urszula Lewandowska

**Affiliations:** 1 Department of Biochemistry, Faculty of Medicine, Medical University of Lodz, Mazowiecka 5, 92-215 Lodz, Poland; milosz.caban@stud.umed.lodz.pl (M.C.); katarzyna.owczarek@umed.lodz.pl (K.O.); wojciech.pawlowski1@stud.umed.lodz.pl (W.P.); karolina.niewinna@umed.lodz.pl (K.N.); 2 Institute of Food Technology and Analysis, Faculty of Biotechnology and Food Science, Lodz University of Technology, Stefanowskiego 2/22, 90-537 Lodz, Poland; justyna.rosicka-kaczmarek@p.lodz.pl (J.R.-K.); karolina.miskiewicz@p.lodz.pl (K.M.); joanna.oracz@p.lodz.pl (J.O.)

**Keywords:** bioavailability, colon, encapsulation, flaxseed polysaccharides, inflammatory bowel diseases, inflammatory response, intestine, polyphenols, spent hop extract

## Abstract

The treatment of inflammatory bowel diseases (IBDs), particularly ulcerative colitis and Crohn’s disease, remains a challenge. As the available therapeutic options have limited efficacy and various side effect, there is a need to identify new inflammatory modulators that can influence IBD. Natural polyphenols and polyphenol-rich extracts have been found to have preventive and therapeutic potential, including various anti-inflammatory effects. In this study, the inhibition of the formation of mediators associated with intestinal inflammation, remodelling, and angiogenesis by the spent hop extract (SHE), a polyphenol-rich extract from *Humulus lupulus* L., and its flaxseed polysaccharide-based encapsulates was examined using tumour necrosis factor alpha (TNF-α)-stimulated human small intestinal epithelial (HIEC-6) and large intestinal epithelial (CCD841CoN) cells. Also, we assessed the activity of the tested agents after in the vitro-simulated gastrointestinal digestion process. SHE strongly inhibited the expression of pro-inflammatory cytokines, mainly IL-1β and TNF-α, as well as the expression and activity of type IV collagenases (MMP-2 and MMP-9); these effects resulted from the suppression of NF-κB, ERK and Akt signalling pathways. We also proved the protective effect of encapsulation process against the reduction in the bioaccessibility of SHE, observed under the influence of digestion process. Our results provide initial evidence on the potential utility of SHE and its encapsulates in IBD.

## 1. Introduction

Inflammatory bowel diseases (IBDs), represented mainly by Crohn’s disease (CD) and ulcerative colitis (UC), are chronic, relapsing disorders which reduce quality of life and constitute significant problems for healthcare [1]. The number of new diagnoses is believed to be growing, and it is estimated that by the year 2030, the prevalence of IBD will have increased 1.8 to 2.6-fold compared to 2010, depending on the region [2]. Pathologically, IBD is characterized by the infiltration of inflammatory cells into the gut and the consequent production of various pro-inflammatory factors, such as cytokines, chemokines, free radicals, that promote oxidative stress, epithelial integrity disruption, and even epithelial barrier damage [3]. The behaviour and natural course of IBD vary between individuals and are highly heterogenous in CD. The location of the disease remains relatively stable. In UC, pathological changes are limited to the colon, starting from the anal margin, whereas in CD, lesions may occur throughout the entire digestive tract, including the small intestine, stomach, esophagus, and even the oral cavity. In addition, CD is characterized by discontinuous, transmural inflammation, while UC involves continuous inflammation confined to the colonic mucosa [4]. An important concern in the context of IBD is colorectal cancer (CRC). Patients with UC and the colonic form of CD (Crohn’s colitis) have an increased risk of developing CRC as a complication of chronic colonic inflammation. Moreover, CRC remains one of the leading causes of mortality and a primary indication for colectomy in patients with IBD [5].

The key to minimizing disease progression, inducing and maintaining disease remission, and improving the patient outcomes is early and efficient treatment. Unfortunately, existing therapeutic options, such as glucocorticosteroids, aminosalicylates, immunomodulators and biological therapies, are associated with many side effects, have limited efficacy, and do not satisfactorily improve quality of life [6]. As such, new, more efficient agents that can prevent exacerbation and mitigate the clinical course of the disease are needed.

It has been found that diet plays an important role in maintaining bowel health in humans and has significant influence on the course of IBD [7,8]. The application of plant-based nutrients is actually growing in popularity. This trend may result from growing awareness of the toxicity of conventional drugs used in IBD therapies, as well as from the demonstrated efficacy of such nutrients in enhancing the therapeutic outcomes of existing treatments. In addition, the consumption of plant-based foods offers advantages over animal-derived and refined products and should be encouraged in patients with IBD. Plant-derived secondary metabolites provide numerous health benefits, including suppressing inflammation, reducing increased intestinal permeability, and helping to prevent the development of IBD-related CRC [9,10]. In particular, significant health benefits have been associated with the consumption of polyphenols, these being common components of fruits and vegetables: furthermore, polyphenolic compounds have been found to demonstrate protective and therapeutic potential in IBD, bestowing various anti-inflammatory or anti-oxidative effects [11]. Plant-derived extracts containing complex mixtures of polyphenols may display synergistic or additive properties compared to the individual compounds. Recent data confirms that the polyphenol-rich extracts can relieve IBD by inhibiting pro-inflammatory cytokine expression, enhancing the epithelial gut barrier, and restoring the composition of the gut microbiota [12]. Unfortunately, polyphenol-rich extracts are characterized by unsatisfactory bioaccessibility and bioavailability after oral administration; however, their effectiveness can be improved using various encapsulation types [13].

One particularly rich source of polyphenols, including phenolic acids, flavonoids and their derivatives, is the extract of spent hops, i.e., the residue obtained from female inflorescences of *Humulus lupulus* Linnaeus [14]. Data indicate that SHE may be a valuable anti-inflammatory agent. SHE attenuated the inflammatory response in lipopolysaccharide (LPS)-stimulated RAW 264.7 mouse macrophages, and exerted a chemopreventive effect against CRC cells by inhibiting angiogenesis, migration and invasion [14,15]. In addition, SHE is inexpensive and readily available, because the source material, i.e., spent hops are generated in large quantities as a waste product during beer production. So far, no studies have assessed the potential of SHE as a treatment for IBD.

The main aim of the present study was to confirm whether SHE is able to inhibit the inflammatory response in an in vitro model of IBD based on human small intestine epithelial cells (HIEC-6) and human colon epithelial cells (CCD841CoN) stimulated with tumour necrosis factor alpha (TNF-α). The study also examines the biological activity of specially designed SHE microcapsules in the same model; the microcapsules were based on bioactive heteropolysaccharides derived from flaxseed. It should be emphasized that this type of carrier in microcapsules has not been used so far. Maltodextrin, chitosan or gum arabic have mainly been used as carriers so far. In addition, to reflect the conditions taking place in humans following consumption, all tested agents were subjected to simulated gastrointestinal digestion and were tested in biological assays. Hence, the study also aimed to determine the impact of the digestion process on the activity of SHE and its encapsulate and assess the efficacy and utility of encapsulation.

## 2. Materials and Methods

### 2.1. SHE

Dried spent hop, a residue from the extraction of hop cones (*Humulus lupulus* L.) obtained by supercritical CO_2_, was supplied by the Fertilizer Research Institute Puławy (Poland). This by-product was ground to fine powder and then extracted by green method ultrasound-assisted extraction (UAE).

### 2.2. Obtaining SHE and Its Encapsulation

In order to obtain extracts from spent hop, 50 g of dried hop cone pomace was weighed into a 1000 mL glass bottle, and a suitable weight was combined with an extraction mixture (50:50 (*v*/*v*) ethanol with water) at a ratio of 1:15 (*m*/*v*) (1 part material: 15 parts extraction mixture). Solid–liquid extraction (SLE) was carried out at room temperature (20 °C) for 1 h using a magnetic stirrer. In the next step, the mixture was extracted using UAE in an ultrasonic bath at 200 W ultrasound (green method extraction) at room temperature (20 °C) for 30 min. After the extraction was complete, the resulting solution was centrifuged at 6000 rpm for 15 min at room temperature (20 °C). The supernatant was decanted, and the resulting precipitate was re-extracted according to the procedure described above. The supernatants obtained from the two extractions were combined. Ethanol from the resulting supernatant was removed using a rotary evaporator until no ethanol condensate was observed in the receiver. The extract obtained after concentration was fixed by sublimation drying (freeze-drying) method. The extraction yield, calculated as the ratio of the freeze-dried extract (SHE) to the initial amount of dried hop cone pomace, was 26%. The resulting lyophilisates were stored at room temperature (20 °C) in tightly closed containers until use.

Encapsulates with the resulting SHE were obtained by spray drying method. Flaxseed polysaccharides (FP) isolated from golden flaxseed seeds were used as a carrier. The extract to carrier ratio was 2:1 (m/m). The spray drying process was carried out using a Buchi Mini Spray Dryer B-290 (BÜCHI Labortechnik AG, Flawil, Switzerland), with the following parameters: temperature at the inlet of the dryer: 110 °C, temperature at the outlet of the dryer: 77 °C. The encapsulation efficiency was calculated based on the ratio between the amount of phenolic compounds identified in the SHE used for encapsulation and detected in the resulting ESHE, and was 92.6%.

### 2.3. Simulated Gastrointestinal Digestion of SHE and Its Encapsulates (ESHE)

The simulated gastrointestinal digestion was performed as described by Kowalska et al. (2022) with some modifications [16]. Briefly, the in vitro digestion, was performed as two phases of static, viz. gastric and intestinal, using two simulating fluids, gastric juice at pH 2 and intestinal juice at pH 7. Firstly, the tested agents (SHE, ESHE and FP) were added to a gastric juice mixture consisting of 0.1 M HCl and pepsin and incubated at 37 °C for 2 h. After incubation the solutions were centrifuged in centrifuge (Centurion Scientific K3 Series) at 3024× *g* for 60 min at 4 °C and a certain portion of the supernatants were decanted and freeze-dried using a freeze-dryer CHRIST Beta 2-8LSCplus (Martin Christ, Gefriertrocknungsanlagen GmbH, Osterode am Harz, Germany), thus obtaining fractions of the tested agents (labelled GSHE, GESHE and GFP), and these can be assumed to reflect the samples located in the human small intestine after gastric digestion. In the next stage, the pH of the residues left after centrifugation were adjusted to 7 and mixed with intestinal juice containing inter alia pancreatin solution and bile salt solution, and subsequently dialyzed using dialysis membranes (MW cut-off > 12.4 kDa) in 0.1 M sodium bicarbonate The mixtures were incubated at 37 °C. After 3 h, the digestive enzymes inactivated and then allowed to cool. The solution was centrifuged in centrifuge (Centurion Scientific K3 Series, Centurion Scientific, Lancing, West Sussex, UK) at 3024× *g* for 10 min at 4 °C, and the supernatants were kept as retentates of the tested agents (viz. RSHE, RESHE and RFP), reflecting the substances located in human large intestine after gastric and intestinal digestion. All digestions were performed in triplicate. A control sample consisting of the gastrointestinal juices, enzymes, and water without the tested samples, was also prepared; this was used to evaluate the possible influence of the digestive enzymes on the results.

### 2.4. Antioxidant Activity of SHE and Encapsulates

#### 2.4.1. Preparation of Extracts from the Digested and Non-Digested SHE, FP and Encapsulates (ESHE) to Determine Antioxidant Activity

Approximately 0.1 g (at an ambient temperature) of digested and non-digested SHE, ESHE and FP was extracted using 10 mL of 70% aqueous methanol solution, at an ambient temperature (25 °C) for 12 h in a rotator (Multi Bio RS-24 BioSan, BioSan SIA, Riga, Latvia). After extraction, samples were centrifuged in centrifuge (Centurion Scientific K3 Series) at 3024× *g* for 15 min at 25 °C. After centrifugation, the supernatants were decanted into clean tubes and stored at −20 °C until analysis.

#### 2.4.2. Determination of Total Polyphenol Content (TPC) by Folin–Ciocalteau Reagent (FCR)

The determination of TPC using the FCR was conducted following Kowalska et al. (2021) with some modification [17]. First, 0.05 mL of the prepared extracts (according to Section 2.4.1) was measured into a 15 mL centrifuge tubes. Then 0.5 mL of Folin–Ciocalteu reagent and 1.5 mL of 20% sodium carbonate solution were added. The centrifuge tubes were filled with distilled water to a volume of 10 mL. The contents of the centrifuge tubes were mixed and incubated at 25 °C for 1 h in the dark. After this time, the absorbance of the solutions was measured on a Shimadzu UV-1800 spectrophotometer (Shimadzu Corporation, Kyoto, Japan) a dose–response curve for gallic acid. A calibration curve was prepared using gallic acid solutions in the concentration range of 0.003–0.099 mg × mL^−1^ (R^2^ = 0.9996). All measurements were performed in triplicate.

#### 2.4.3. Determination of Flavonoid Content

The flavonoid content was determined using a spectrophotometric method described by El Hariri et al. (1991) and Gumul et al. (2023) with some modification [18,19]. First, 0.05 mL of the prepared extracts (according to Section 2.4.1) was measured into a 7 mL tubes. Then, 0.15 mL of 15% NaNO_2_ solution, 0.15 mL of 10% AlCl_3_ solution and 2 mL of 4% NaOH solution were added. The tubes were filled with distilled water to a volume of 5 mL. The contents of the tubes were mixed and incubated at 25 °C for 15 min in the dark. After this time, the absorbance of the solutions was measured on a Shimadzu UV-1800 spectrophotometer at a wavelength of λ = 420 nm against distilled water. The results were reported as mg of quercetin equivalents (QE) per 100 g of SHE and its encapsulates with reference to a calibration curve for quercetin. A calibration curve was prepared using quercetin solutions in the concentration range of 0.001–0.03 mg × mL^−1^ (R^2^ = 0.9994). All measurements were performed in triplicate.

#### 2.4.4. Determination of Antioxidant Activity Using ABTS+ Assay

Antioxidant capacity of the SHE and its encapsulates were determined by ABTS assay according to the method described by Kowalska et al. (2021) with some modifications [17]. First, the ABTS radical stock solution was prepared by mixing 7 mM ABTS with 2.45 mM potassium persulfate (5 mL of ABTS plus 5 mL of potassium persulphate 2.45 mM). The mixture was then incubated for 16 h in the dark at 20 °C. Then, the working solution of ABTS+ cation radical was prepared by diluting the stock solution with distilled water to obtain an absorbance of 0.700 ± 0.002 at a wavelength of λ = 734 nm (Shimadzu Spectrophotometer UV-Visible 1800, Shimadzu Corporation, Kyoto, Japan). First, 0.01 mL of SHE and ESHE (prepared according to the method described in Section 2.4.1.) was measured into 7 mL test tubes. Then, 4 mL of 7 mM ABTS+ cation radical working solution was added to the test tubes, and each test tube was filled with distilled water to a volume of 4.5 mL. At the same time, a control sample was prepared consisting of 0.5 mL of distilled water and 4 mL of 7 mM ABTS+ cation radical working solution. The contents of the test tubes were mixed and incubated at 25 °C for 20 min without access to light. After this time, the absorbance was measured on a Shimadzu UV-1800 spectrophotometer (Shimadzu Corporation, Kyoto, Japan) at a wavelength of λ = 734 nm against distilled water. The absorbance values measured for the actual samples were corrected by the absorbance value measured for the control sample. The measurements were carried out in triplicate and the results were expressed as millimoles of Trolox equivalents (TE) per 100 g of SHE and its encapsulates (mM TE 100 g^−1^) with reference to a calibration curve for Trolox. A calibration curve was prepared using Trolox solutions in the concentration range of 5.01 × 10^−6^–6.01 × 10^−5^ mM (R^2^ = 0.9989).

#### 2.4.5. Determination of Antioxidant Activity Using the DPPH Assay

The DPPH free radical scavenging capacity was determined as described by Kim (2020) with some modification [20]. First, 0.02 mL of extracts were measured into 7 mL test tubes and the contents of the test tubes were supplemented with ethanol to a volume of 1 mL. Then, 4 mL of 0.12 mM ethanolic solution of DPPH radical were added to the test tubes. The contents of the test tubes were mixed and then incubated at 25 °C without access to light for 30 min. After the incubation, the absorbance of the samples was measured on a Shimadzu UV-188 spectrophotometer (Tokyo, Japan) at a wavelength of λ = 517 nm against ethanol. The absorbance values measured for the actual samples were corrected by the absorbance value of the control sample, which consists of 1 mL of distilled water and 4 mL of 0.12 mM ethanolic solution of DPPH radical. The measurements were carried out in triplicate and the results were expressed as millimoles of Trolox equivalents (mM TE) per 100 g of SHE and its encapsulates (mM TroloxTE 100 g^−1^) with reference to a calibration curve for Trolox. A calibration curve was prepared using Trolox solutions in the concentration range of 2.50 × 10^−5^–45.09 × 10^−5^ mM (R^2^ = 0.9994).

#### 2.4.6. Determination of Reducing Power Potential by the Method of FRAP Assay

The Reducing Power Potential by the Method of FRAP Assay was determined as described by Gumul et al. (2023) with some modification [19]. First, the FRAP reagent was prepared. To prepare the FRAP reagent, 300 mM acetate buffer (pH 3.6), 40 mM 2,4,6-tri(2-pyridyl)-triazine (TPTZ) (dissolved in 40 mM HCl), and 20 mM ferric chloride (dissolved in water) were mixed in a ratio of 10:1:1 (*v*/*v*), respectively. First, 0.01 mL of spent hop and spent hop encapsulates extracts were measured into 7 mL samples. Then, 1.2 mL of FRAP reagent and 2 mL of distilled water were added to each test tube. A control sample was prepared in parallel with the actual tests. For this purpose, 0.01 mL of distilled water was measured into a 7 mL test tube, and then 1.2 mL of FRAP reagent and 2 mL of distilled water were added. The whole was mixed vigorously and incubated at 25 °C for 4 min in the dark. After this time, absorbance was measured on a Shimadzu UV-1800 spectrophotometer (Tokyo, Japan) at a wavelength of λ = 593 nm. The results were expressed as mmol of Fe^2+^ per 100 g of SHE and its encapsulates, with reference to a calibration curve for iron (II) sulphate. A calibration curve was prepared using iron (II) sulphate solutions in the concentration range of 10.03 × 10^−3^–200.38 × 10^−3^ mM (R^2^ = 0.999).

#### 2.4.7. Qualitative and Quantitative Analysis of Phenolic Compounds in Extract by UHPLC-DAD

The content of phenolic compounds of SHE and its encapsulates was determined according to Oracz et al. (2022) with our own modification [21], using an HPLC  +  Ultimata 3000 chromatograph by Dionex, with a UV-Vis detector, in an Accucore C18 column (2.6 µm, 150 mm × 2.1 mm i.d. Thermo Scientific, Thermo Fisher Scientific Inc., Waltham, Massachusetts, United States) thermostated in 28 °C with 10 µL injection volume. A gradient elution mode was used: 1% formic acid in water (phase A) and acetonitrile (phase B), at a flow rate of 0.35 mL min^−1^. Separation was performed with the following gradient programme: 0 min, 1.0% B; 8 min, 5.0% B; 15 min, 8.0% B; 20 min, 10.0% B; 25 min, 15.0% B; 35 min, 20.0% B; 40 min, 25% B; 50 min, 90.0% B; 53 min, 90.0% B; 58 min, 1.0% B; 65 min, 1.0% B. Standard solutions were used for the identification of phenolic compounds, and determinations were performed by measuring absorbance at several wavelengths: hydroxybenzoic acids—280 nm, hydroxycinnamic acids—320 nm, flavonols—365 nm and prenylflavonoids—270 nm. Chromeleon 6.8.1 Chromatography Data System software was used to control, record and analyze the obtained results. The detailed data about detection limits, linearity ranges and recovery percentages for individual phenolic compounds determined by UHPLC-DAD was presented in Supplementary Table S1.

### 2.5. Surface Morphology Assessment with Scanning Electron Microscopy

The surface morphology and microstructure of the obtained encapsulates (ESHE) were studied by scanning electron microscopy (SEM) using a Jeol-JCM-6000 scanning electron microscope (JEOL JCM-6000 Scanning Electron Microscope (SEM), JEOL Ltd., Akishima, Tokio, Japan). The examined samples were sputter-coated with gold under vacuum without any noble gas. Images were recorded at differences in acceleration potentials ranging from 5 kV to 10 kV mode.

### 2.6. Cell Culture

Two cell lines, HIEC-6 cells derived from human small intestine epithelium and CCD841CoN cells derived from human large intestine epithelium, were obtained from the American Type Culture Collection (ref: CRL-3266 and CRL-1790, respectively). The HIEC-6 cells were cultured in OptiMEM 1 Reduced Serum Medium (Gibco^®^, Grand Island, NY, USA) supplemented with 4% fetal bovine serum (FBS), 20 mM HEPES, 10 mM GlutaMAX (Gibco^®^), 10 ng/mL epidermal growth factor (EGF) (Sigma-Aldrich, St. Louis, MO, USA), 50 U/mL penicillin and 50 µg/mL streptomycin.

The CCD841CoN cells were cultured in Eagle’s Medium Essential Medium (EMEM) (Gibco^®^, Grand Island, NY, USA) supplemented with 10% FBS, 2 mM L-glutamine (Gibco^®^), 50 U/mL penicillin and 50 µg/mL streptomycin. The quantity of FBS was reduced to 3% during incubation with the tested agents.

In all cases the cells were grown at 37 °C in a humidified atmosphere containing 5% CO_2_. The cells were seeded in such quantities that the confluence did not exceed 80% in the control wells at the end of the experiment. Additionally, bevacizumab (anti-vascular endothelial growth factor A antibody) (250 μg/mL) (Avastin^®^, Roche Pharma AG, Grenzach-Wyhlen, Germany), budesonide (glucocorticosteroid) (1 μM) (Sigma-Aldrich) or Bay 11-7082 (an inhibitor of NF-κB) (2.5 μM) (Sigma-Aldrich) were used as positive controls in individual experiments. Budesonide was chosen as a positive control in most experiments, as it is recommended for the treatment of mild to moderate UC and mild to moderate ileocaecal form of CD. TNF-α (30 ng/mL) (Sigma-Aldrich) was used to stimulate the inflammatory response. For all bioassays, the tested agents were dissolved in medium. The agents formed by gastric digestion (GSHE, GESHE, GFP) were tested on the HIEC-6 cells and those formed by intestinal digestion (RSHE, RESHE, RFP) on the CCD841CoN cells to reflect their contact in real life.

### 2.7. Cell Viability Assay

The effect of the tested agents on the viability of the HIEC-6 and CCD841CoN cells was evaluated by 3-(4,5-dimethylthiazol-2-yl)-2,5-diphenyltetrazolium bromide (MTT) (Sigma Aldrich) reduction assay. In a 96-well plate, 8 × 10^3^ cells per well were seeded in a growth medium containing for 24 h. Following this, the medium was replaced with one containing the tested agents at various concentrations for another 24 h. Then, 20 µL of MTT solution (5 mg/mL) was added to each well. After two hours, the medium with MTT was precisely discarded and 100 µL dimethyl sulfoxide (DMSO) was added to each well. Finally, the samples were shaken for 15 min at the room temperature, and their absorbance at 595 nm optical density (OD) was measured using an iMarkTM microplate reader (BioRad Labs. Inc., Des Plaines, IL, USA). The results are given as the means of three repetitions from three independent experiments.

### 2.8. RNA Extraction and Quantitative Real Time PCR (q-PCR)

After 24 h incubation with standard culture medium, the cells were stimulated with TNF-α for 16 h with or without the tested agents. The procedures for RNA isolation, cDNA synthesis and q-PCR are given by Caban et al. (2022) [15]. Total RNA extraction was performed using TRIzol^®^ reagent (Invitrogen™, Carlsbad, CA, USA). The mRNA expression of the following specific target genes was determined: IL-1β (Hs01555413_m1), IL-6 (Hs00174131_m1), TNF (Hs00174128_m1), MMP-2 (Hs01548727_m1), MMP-9 (Hs00234579_m1), KDR (Hs00911700_m1), HIF1A (Hs00153153_m1), RELA (Hs00153294_m1). All analyses used probes from TaqMan^®^ Gene Expression Assays (Applied Biosystems, Waltham, MA, USA). cDNA synthesis was performed using Maxima^®^ First Strand cDNA Synthesis Kit for RT-qPCR. qPCR was performed utilizing a qPCR Master Mix (FastStart Essential DNA Probes Master, Roche, Basel, Switzerland) with a LightCycler^®^ 96 System thermocycler (Roche, Basel, Switzerland). Glyceraldehyde 3-phosphate dehydrogenase (GAPDH; Hs02758991_g1) (Applied Biosystems, Waltham, MA, USA) was used as a reference gene for qPCR. The threshold cycle (Ct) values for studied genes were normalized to the housekeeping gene GAPDH Ct values and the relative amounts of each gene was calculated using the 2^−ΔΔCt^ method.

### 2.9. Protein Samples Preparation and Western Blot Analysis

Firstly, HIEC-6 and CCD841CoN cells were seeded in growth medium in a six-well plate, and left to incubate for 24 h. After this time, the cells were suspended in the medium containing TNF-α, the tested agents, Bay 11-7082 or budesonide, or none of them, and incubated for a further 6 h.

Cell lysates were prepared using a Mammalian Cell Lysis Kit (Sigma-Aldrich) followed by Bradford reagent (Bio-Rad Labs. Inc., Des Plaines, CA, USA): this was used to determine the protein content of the samples. Subsequently, the protein was separated on a MiniPROTEAN^®^ TGXTM gel (Bio-Rad Labs. Inc., Des Plaines, CA, USA), 40 µg of protein in each well. The gels were then subjected to Western blotting as described previously [14]. Immunoblotting was performed using the following antibodies: β-actin (sc-47778), Akt1 (sc-5298), p-Akt1 (sc-293125), ERK 1/2 (sc-514302), p-ERK (sc-7383), NF-kB p65 (sc-8008), p-NF-kB p65 (sc-135769) and m-IgGk BP-HRP (sc-516102). All antibodies were supplied by Santa Cruz Biotechnology (Dallas, TX, USA).

### 2.10. Enzyme-Linked Immunosorbent Assay (ELISA)

HIEC-6 and CCD841CoN cells were seeded in six-well plates and allowed to grow in medium for 24 h. Following this, the cells were suspended in medium with or without TNF-α, tested agents, budesonide and bevacizumab. After 24 h of incubation, the medium was collected and subjected to ELISA to determine IL-1β and IL-6 protein expression. TNF-α and VEGFR2 protein expression was evaluated using protein cell lysates. Briefly, the medium was removed and cell lysis performed using RIPA buffer mixed with protease inhibitor cocktail (Sigma-Aldrich). Then, the protein content of the cell lysates was determined using Bradford reagent. Equal quantities of cell lysates were used to test for different proteins. The level of each protein was determined with the following ELISA kits in accordance with the manufacturer’s instructions: human IL-1β (Cloud-Clone USCN Life Science, Wuhan, China), human IL-6 (Cloud-Clone USCN Life Science, Wuhan, China), human TNF-α (RayBiotech, Inc., Norcross, GA, USA) and human VEGFR2 (RayBiotech, Inc., Norcross, GA, USA). In all cases, the optical density of the samples was measured at 450 nm using a microplate reader.

### 2.11. Zymography Assay

HIEC-6 and CCD841CoN cells were seeded in 96-well plates in medium and incubated for 24 h: following this, the medium was replaced by medium containing TNF-α, budesonide and the tested agents and incubated for 24 h. For electrophoresis, 10 µL of conditioned media samples were mixed with 5 µL of non-reducing sample buffer and separated by 10% PAGE embedded with gelatine. MMP-2 and MMP-9 were then renatured by washing with 2% Triton X-100 and rinsing with 50 mmol/L Tris-HCl (pH 7.4). The gels were incubated in developing buffer containing 1% Triton X-100, 50 mmol/L Tris-HCl (pH 7.4) and 5 mmol/L CaCl_2_ for at least 21 h at 37 °C.

The activities of the gelatinases were determined by staining with a solution containing 0.1% Amido Black, 7% acetic acid and 20% ethanol: the results were photographed using an Olympus camera (Olympus Corp., Tokyo, Japan). Gelatinase activity was visualized as a transparent band against the dark blue background of the Amido Black-Stained Slab gels. Densitometry analysis was conducted by the GelDoc ™EQ system with Quantity One software version 4.4.1 (Bio-Rad Laboratories, Inc., Hercules, CA, USA).

### 2.12. Statistical Analysis

The statistical analysis was performed by one-way ANOVA followed by the Newman–Keuls post hoc test using GraphPad Prism 9 (La Jolla, CA, USA). Data are expressed as mean ± SEM. *p*-values < 0.05 were considered to be statistically significant. Experiments were performed in at least triplicate. Detailed data about statistical significance are given in the figure legends.

## 3. Results

### 3.1. The Composition of the Tested Agents

The highest levels of phenolic compounds were found for SHE (879.73 ± 3.21 mg/100 g extract), followed by ESHE (543.03 ± 1.04 mg/100 g extract). The digestion process reduced the phenolic compounds content. In the detected phenolics, five groups were identified: hydroxycinnamic acids, hydroxybenzoic acids, flavan-3-ols, flavonols, and prenylflavonoids.

In all tested agents, except FP and its digested forms, the dominant phenolic compounds were flavonols (Table 1). The predominant compound was quercetin-3-O-glucoside in SHE, RSHE, ESHE and RESHE, and quercetin-3-O-rutinoside in GSHE and GESHE. In contrast, ferulic acid dominated in FP, GFP and RFP. A more detailed description of the composition of flavonols, hydroxycinnamic acids, hydroxybenzoic acids and prenylflavonoids is given in Table 2, Table 3, Table 4 and Table 5, respectively. The highest TPC (4418.48 ± 1.91 mg GAE/100 g) and TFC (2295.71 ± 1.56 mg QE/100 g) values were noted in SHE, followed by ESHE (TPC 2758.32 ± 1.98 mg GAE/100 g and TFC 1389.72 ± 1.65 mg QE/100 g) (Table 6). Interestingly, higher TPC and TFC were noted for GESHE and RESHE than GSHE and RSHE (Table 6), indicating that the encapsulation process may protect polyphenols against digestion.

### 3.2. Assessment of the Surface Morphology of Microcapsules

**Figure 1:**
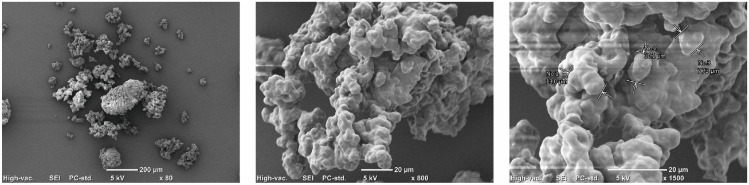
Microstructure of encapsulated SHE (ESHE) with polysaccharides isolated from flaxseed as a coating material. Photos from scanning electron microscopy with various magnification (80×, 800× and 1500×).

The morphological structure of the tested encapsulates (ESHE), as revealed by micrographic analysis, is given in Figure 1. The mean diameter of the encapsulate particles was 10.3 μm. The encapsulates were characterized by optimal microcapsule morphology, i.e., a uniform, spherical shape, and a smooth surface. The obtained SEM microscopic images for the analyzed preparations indicate that the microcapsule particles tended to form agglomerate-like arrangements, thus indicating a tendency for the encapsulated particles to cohere to one another.

### 3.3. Antioxidant Activity

The study also examined the differences in DPPH and ABTS radical scavenging and FRAP values between treatments. The SHE group demonstrated significantly higher DPPH (34.07 ± 3.29 mM TE/100g) and ABTS radical scavenging activities (52.91 ± 1.34 mM TE/100g) compared to ESHE. Also, DPPH and ABTS activities were significantly reduced by simulated digestion: GSHE demonstrated approximately 96% and 75% lower activity compared to SHE, and RSHE demonstrated approximately 99% and 78% lower activity by comparison.

Interestingly, following digestion, higher DPPH and ABTS values were noted for ESHE compared to GESHE and RESHE compared to ESHE, indicating that encapsulation process had a protective effect on the antioxidant activities of SHE. More specifically, compared to ESHE, DPPH activities were about 65% lower for GESHE and 92% lower for RESHE. ABTS was 32% lower for GESHE, and 23% higher for RESHE. The FRAP values of the encapsulated digested extracts (viz. GESHE and RESHE) were higher than those of the non-encapsulated extracts (viz. GSHE and RSHE): these respective values ranged from 12.67  ±  0.38 to 13.66  ±  0.40 mM Fe^2+^/100 g for GESHE and RESHE (encapsulated) and from 5.19  ±  0.30 to 9.26  ±  0.31 mM Fe^2+^/100 g for GSHE and RSHE (non-encapsulated) (Table 6).

### 3.4. Effect of the Tested Agents on Viability of HIEC-6 and CCD841CoN Cells

The HIEC-6 cells were treated with SHE, GSHE, ESHE, GESHE, FP and GFP, while CCD841CoN cells were treated with SHE, RSHE, ESHE, RESHE, FP and RFP. All cultures were treated for 24 h with the tested agents following concentrations: 50, 100, 150, 200, 250, 300, 400 and 500 µg/mL. Control samples were incubated without any agent.

**Figure 2:**
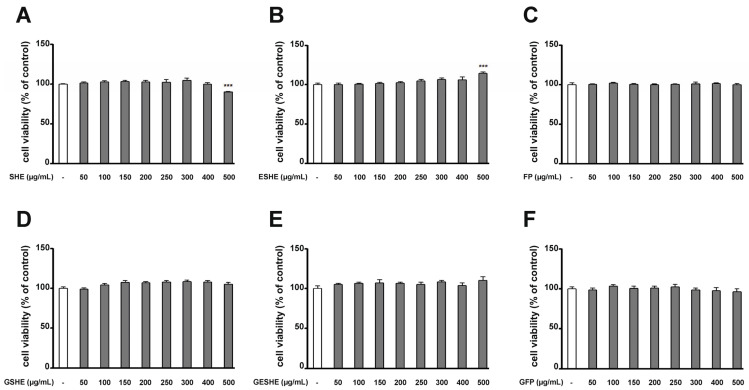
The effect of tested agents ((**A**)—SHE; (**B**)—ESHE; (**C**)—FP; (**D**)—GSHE; (**E**)—GESHE; (**F**)—GFP) on cell viability in human small intestine epithelial cells HIEC-6. The viability of HIEC-6 cells was determined by MTT assay after incubation with indicated concentrations for 24 h. Each value represents the mean value ± SEM, n = 3 independent experiments (each experiment was carried out in triplicate). The significance of differences between means: *** *p* < 0.001 versus control (untreated cells).

**Figure 3:**
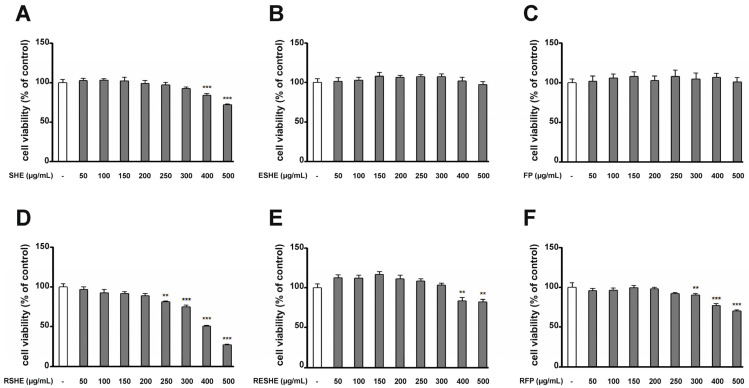
The effect of the tested agents ((**A**)—SHE; (**B**)—ESHE; (**C**)—FP; (**D**)—RSHE; (**E**)—RESHE; (**F**)—RFP) on cell viability in human colon epithelial cells CCD841CoN. The viability of CCD841CoN cells was determined by MTT assay after incubation with indicated concentrations for 24 h. Each value represents the mean value ± SEM, n = 3 independent experiments (each experiment was carried out in triplicate). The significance of differences between means: ** *p* < 0.01, *** *p* < 0.001 versus control (untreated cells).

In the HIEC-6 cells, after 24 h incubation, only 500 µg/mL concentrations of SHE and ESHE altered cell viability (Figure 2). In turn, in CCD841CoN cells (Figure 3), statistically significant changes in cell viability were noted at concentrations of 400 and 500 µg/mL of SHE (Figure 3A), 250, 300, 400 and 500 µg/mL of RSHE (Figure 3D) and 300, 400 and 500 µg/mL of RFP (Figure 3F).

Any variant which demonstrated any significant change in cell viability, based on the MTT test, was taken for further study. The 200 and 400 µg/mL variants were taken for further analyses for the HIEC-6 cells, and the 100 and 200 µg/mL variants for the CCD841CoN cells.

### 3.5. Effect of the Tested Agents on Expression of Pro-Inflammatory Cytokines (IL-1β, IL-6, TNF-α)

**Figure 4:**
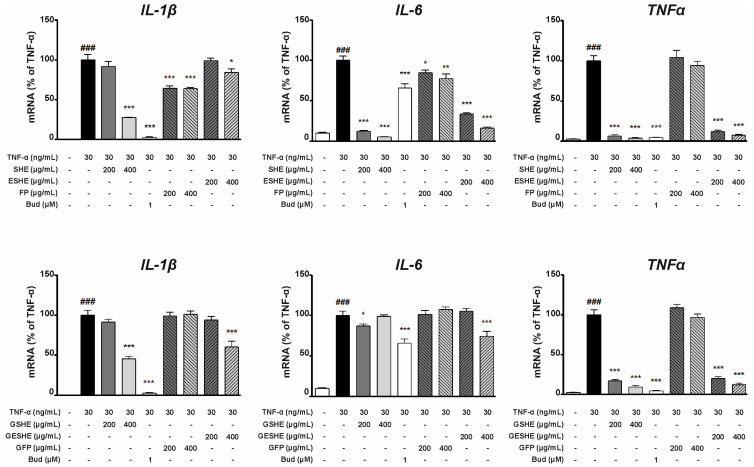
Effects of the tested agents on TNF-α-induced mRNA expression of pro-inflammatory cytokines in HIEC-6 cells. TNF-α-stimulated (30 ng/mL) HIEC-6 were treated with the tested agents (200, 400 µg/mL). The mRNA levels of IL-1β, IL-6 and TNF-α were measured by quantitative polymerase chain reaction (Q-PCR) after 16 h treatment. Budesonide (Bud, 1 µM) was used for comparison. Each value on the graphs represents the mean value ± SEM, n = 3 independent experiments. Significance of differences between means: ^###^ *p* < 0.001 versus control cells, * *p* < 0.05, ** *p* < 0.01, *** *p* < 0.001 versus TNF-α-stimulated cells.

In the TNF-α-stimulated HIEC-6 cells, SHE inhibited the mRNA expression of IL-1β, IL-6 and TNF-α. Interestingly, 200 µg/mL and 400 µg/mL SHE decreased the gene expression of IL-6 more effectively than budesonide (by 88% and 94.7%, respectively), as did 400 µg/mL (by 96.2%) compared to TNF-α. In contrast, GSHE significantly reduced IL-1β mRNA expression at 400 µg/mL (by 54.5%), as well as TNF-α mRNA expression at 200 µg/mL (by 82.9%) and 400 µg/mL (by 90.6%). In turn, 200 and 400 µg/mL ESHE lowered the expression of IL-6 by 66.5% and 84%, respectively, and TNF-α by 87.9% and 92.4%, respectively. ESHE treatment yielded only slight changes in IL-1β mRNA expression. However, GESHE reduced the gene expression of TNF-α in a concentration-dependent manner and lowered the expression of IL-1β and IL-6 at higher tested concentration (400 µg/mL). FP decreased IL-1β and IL-6 mRNA expression in a concentration-dependent manner without any impact on TNF-α gene expression. GFP did not alter the mRNA expression of pro-inflammatory cytokines (Figure 4).

**Figure 5:**
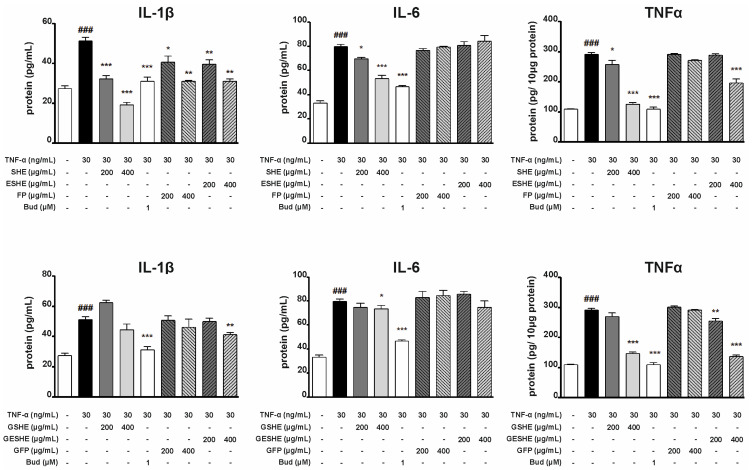
Effects of the tested agents on the TNF-α-induced protein expression of pro-inflammatory cytokines in HIEC-6 cells. TNF-α-stimulated (30 ng/mL) HIEC-6 were treated with the tested agents (200, 400 µg/mL). The protein expression of IL-1β, IL-6 and TNF-α in TNF-α-stimulated HIEC-6 cells was examined by an enzyme-linked immunosorbent assay after 24 h treatment. Budesonide (Bud, 1 µM) was used for comparison. Each value on the graphs represents the mean value ± SEM, n = 3 independent experiments. Significance of differences between means: ^###^ *p* < 0.001 versus control cells, * *p* < 0.05, ** *p* < 0.01, *** *p* < 0.001 versus TNF-α-stimulated cells.

In addition, in both the HIEC-6 and CCD841CoN cell cultures, IL-1β and IL-6 protein expression was determined using cell supernatants, and for TNF-α using cell lysates. In HIEC-6 cells, the SHE treatment suppressed protein production in a concentration-dependent manner for all cytokines. In contrast, GSHE significantly diminished only the protein expression of TNF-α in the 400 µg/mL variant: treatment reduced by 145.6 pg/10 μg protein compared to the TNF-α-stimulated sample. In contrast, ESHE significantly reduced the IL-1β protein production of IL-1β in a concentration-dependent manner (11.5 pg/mL for 200 µg/mL and 20.2 pg/mL for 400 µg/mL), compared to the variant stimulated by TNF-α alone. Also, 400 µg/mL ESHE decreased the TNF-α protein expression by 95.6 pg/10 μg protein compared to the TNF-α-stimulated sample. ESHE treatment did not influence IL-6 protein production. GESHE significantly inhibited the protein expression of TNF-α in a concentration-dependent manner, and to a greater degree than its non-digested form, i.e., ESHE. FP only reduced the protein production of IL 1-β by 10.6 pg/mL (200 µg/mL) and 20.1 pg/mL (400 µg/mL); however, the digested form (GFP) did not cause any significant changes in protein level for any cytokine (Figure 5).

**Figure 6:**
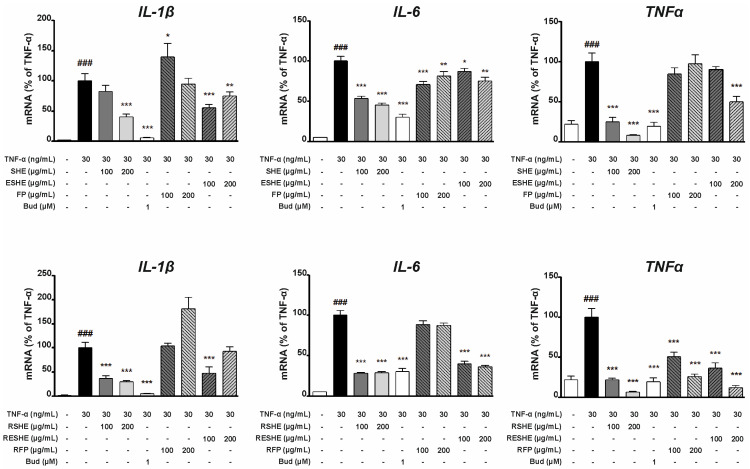
Effects of the tested agents on TNF-α-induced mRNA expression of pro-inflammatory cytokines in CCD841CoN cells. TNF-α-stimulated (30 ng/mL) CCD841CoN were treated with the tested agents (100, 200 µg/mL). The mRNA levels of IL-1β, IL-6 and TNF-α were measured by quantitative polymerase chain reaction (Q-PCR) after 16 h treatment. Budesonide (Bud, 1 µM) was used for comparison. Each value on the graphs represents the mean value ± SEM, n = 3 independent experiments. Significance of differences between means: ^###^ *p* < 0.001 versus control cells, * *p* < 0.05, ** *p* < 0.01, *** *p* < 0.001 versus TNF-α-stimulated cells.

In CCD841CoN cells, SHE inhibited the mRNA expression of all assessed pro-inflammatory cytokines in a concentration-dependent manner. At concentrations of 100 and 200 µg/mL, IL-6 expression was reduced by 46.7% and 54.6%, and TNF-α mRNA expression was reduced by 74.9% and 91.9%, respectively, compared to variants not exposed to SHE. Interestingly, the digested form of SHE, RSHE, caused greater inhibition for all cytokines than the non-digested form. A similar relationship was observed for ESHE and RESHE, except for IL-1β expression. This suggest that the microcapsules may protect against digestion, thus maintaining the activity of the tested agents. In detail, 100 and 200 µg/mL RESHE markedly reduced IL-6 mRNA expression by 60.4% and 64.4%, and TNF-α mRNA expression by 63.6% and 87.9% compared to the TNF-α-stimulated alone (Figure 6).

**Figure 7:**
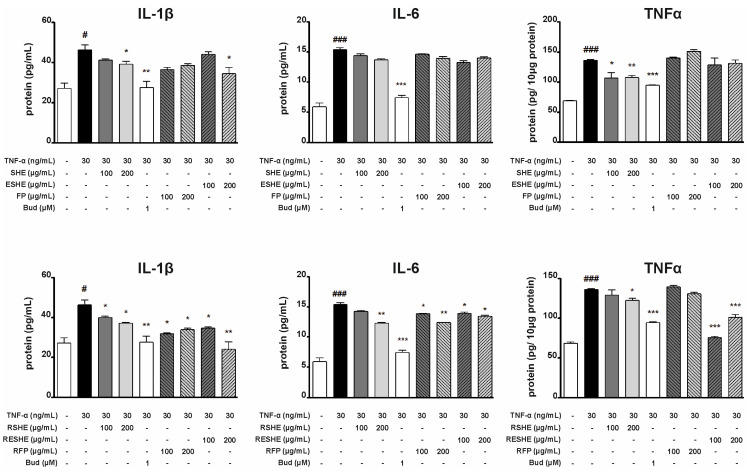
Effects of the tested agents on TNF-α-induced protein expression of pro-inflammatory cytokines in CCD841CoN cells. TNF-α-stimulated (30 ng/mL) CCD841CoN were treated with the tested agents (100, 200 µg/mL). The protein expression of IL-1β, IL-6, and TNF-α in TNF-α-stimulated CCD841CoN cells was examined by an enzyme-linked immunosorbent assay after 24 h treatment. Budesonide (Bud, 1 µM) was used for comparison. Each value on the graphs represents the mean value ± SEM, n = 3 independent experiments. Significance of differences between means: ^#^ *p* < 0.05, ^###^ *p* < 0.001 versus control cells, * *p* < 0.05, ** *p* < 0.01, *** *p* < 0.001 versus TNF-α-stimulated cells.

While SHE decreased the protein production of IL-1β and TNF-α, the other non-digested tested agents (ESHE, FP) did not cause significant changes in protein levels (except for IL-1β at 200 µg/mL of ESHE). However, RESHE inhibited IL-1β and IL-6 protein production in a concentration-dependent manner, as well as the TNF-α protein expression, again demonstrating that encapsulation may improve the efficacy of the tested agents. In turn, RSHE caused slight inhibition of IL-1β and IL-6 protein production, as well as TNF-α protein expression (Figure 7).

### 3.6. Effect of the Tested Agents on Expression and Activity of Gelatinases (MMP-2, MMP-9)

**Figure 8:**
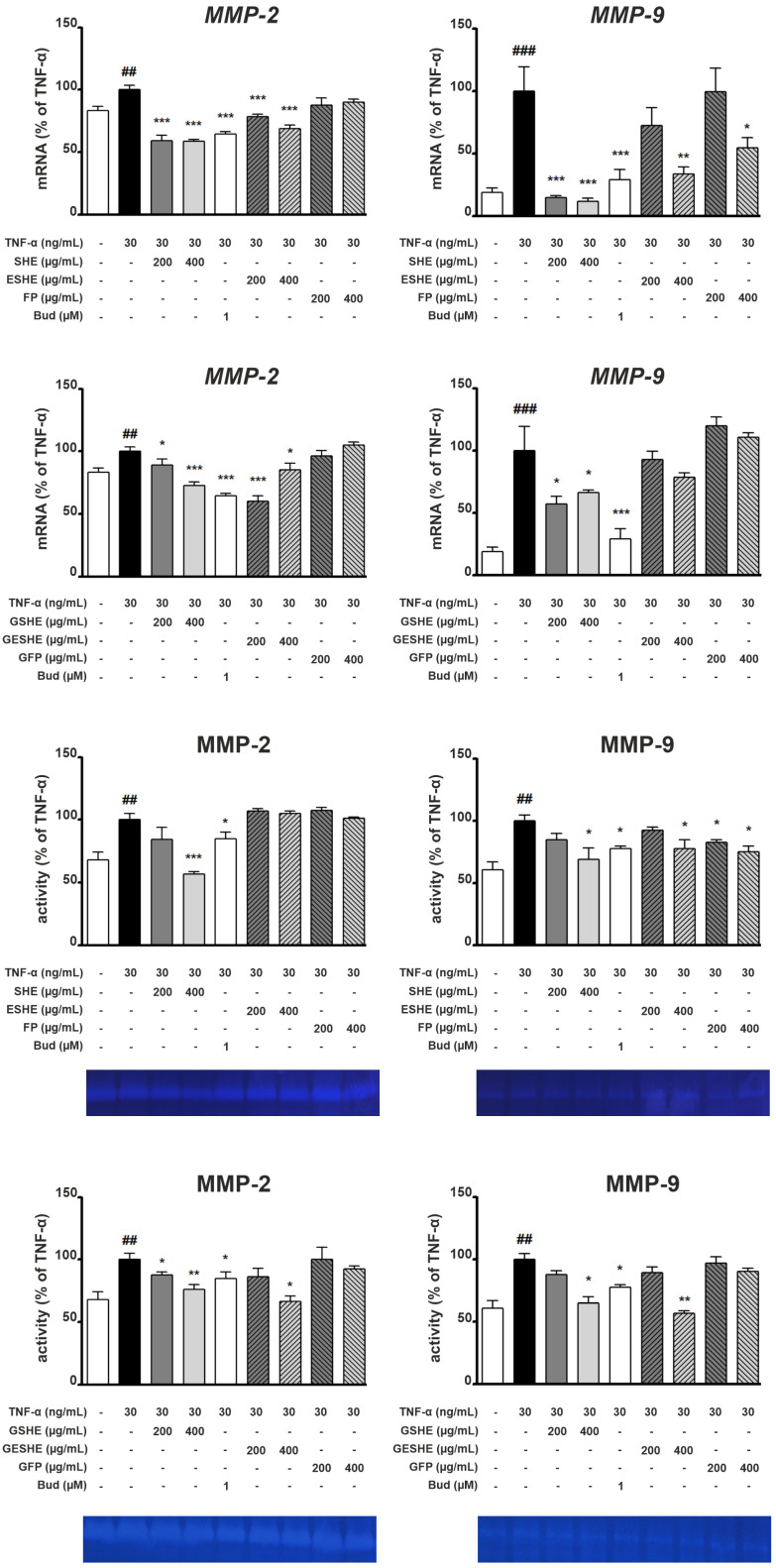
Effects of the tested agents on the mRNA expression and activity of MMP-2/MMP-9 in TNF-α-stimulated HIEC-6 cells. TNF-α-stimulated (30 ng/mL) HIEC-6 were treated with the tested agents (200, 400 µg/mL). The mRNA levels were measured by quantitative polymerase chain reaction (Q-PCR) after 16 h treatment. Zymographic analysis of the media was carried out after 24 h incubation with the tested agents. Representative zymograms were obtained after 24-h incubation. Budesonide (Bud, 1 µM) was used a positive control. Each value on the graphs represents the mean value ± SEM, n = 3 independent experiments. Significance of differences between means: ^##^ *p* < 0.01, ^###^ *p* < 0.001 versus control (untreated cells), * *p* < 0.05, ** *p* < 0.01, *** *p* < 0.001 versus TNF-α-stimulated cells.

Type IV collagenase gene expression and activity were induced in both cell lines using TNF-α. In HIEC-6 cells, the treatment with SHE and ESHE significantly reduced MMP-2 mRNA expression for all tested concentrations; the same was noted for their digested forms, GSHE and GESHE. SHE significantly decreased MMP-2 mRNA expression by 40.6% (200 µg/mL) and 41.1% (400 µg/mL) compared to the TNF-α-activated cells, and these effects were stronger than for GSHE (by 10.6% and 27.1%). In turn, ESHE reduced MMP-2 mRNA expression by 21.5% and 31.1% for 200 and 400 µg/mL, respectively, while GESHE reduced it by 39.8% and 14.6%. MMP-2 activity was reduced by treatment with 400 µg/mL of SHE (by 43%), 200 µg/mL (by 12.5%) and 400 µg/mL (by 24%) of GSHE and by 400 µg/mL of GESHE (by 33.5%). Incubation with FP or GFP did not affect mRNA expression or activity of MMP-2 (Figure 8).

SHE treatment reduced MMP-9 mRNA expression by 85.1% (200 µg/mL) and by 88.3% (400 µg/mL) compared to the TNF-α-stimulated sample: in contrast budesonide treatment caused a decrease by 70.3%. In addition, SHE demonstrated stronger inhibition of MMP-9 mRNA expression than GSHE, which reduced expression by 42.6% (200 µg/mL) and 33.3% (400 µg/mL), respectively (Figure 8). The strongest MMP-9 inhibition (by 43%) was observed for GESHE at 400 µg/mL. Also, this effect was more effective than ESHE, showing that encapsulation process may protect the SHE contained in microcapsules.

**Figure 9:**
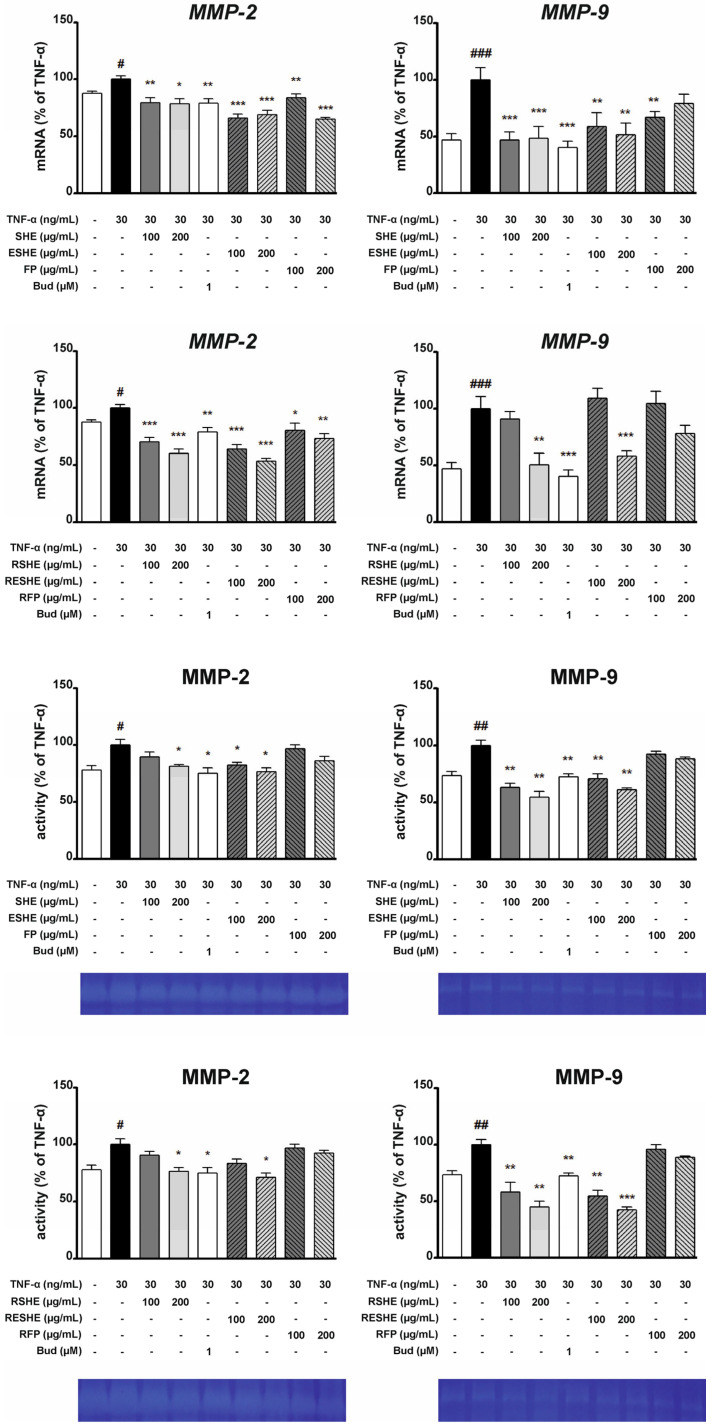
Effects of the tested agents on the mRNA expression and activity of MMP-2/MMP-9 in TNF-α-stimulated CCD841CoN cells. TNF-α-stimulated (30 ng/mL) CCD841CoN were treated with the tested agents (100, 200 µg/mL). The mRNA levels were measured by quantitative polymerase chain reaction (Q-PCR) after 16 h treatment. Zymographic analysis of the media was carried out after 24 h incubation with the tested agents. Representative zymograms were obtained after 24 h incubation. Budesonide (Bud, 1 µM) was used a positive control. Each value on the graphs represents the mean value ± SEM, n = 3 independent experiments. Significance of differences between means: ^#^ *p* < 0.05, ^##^ *p* < 0.01, ^###^ *p* < 0.001 versus control (untreated cells), * *p* < 0.05, ** *p* < 0.01, *** *p* < 0.001 versus TNF-α-stimulated cells.

In CCD841CoN cells, SHE and RSHE treatment caused a concentration-dependent reduction in MMP-2 mRNA expression and activity (Figure 9). It is worth emphasizing that FP and RFP suppressed MMP-2 mRNA expression. In addition, RESHE inhibited the mRNA expression of MMP-2 more strongly than SHE, ESHE and RSHE, at the same concentration. This may also indicate that encapsulation has protective effect, and the RSHE and RFP have an additive effect. RESHE suppressed MMP-2 expression by 36% (100 µg/mL) and 46.3% (200 µg/mL) compared to TNF-α-activated CCD841CoN cells (Figure 9). In turn, MMP-2 activity was suppressed by ESHE, and by higher concentrations of SHE, RSHE and RESHE. Moreover, 200 µg/mL of RESHE showed stronger inhibitory properties than budesonide (decrease to 75%) (Figure 9).

In TNF-α-induced CCD841CoN cells, 200 µg/mL SHE and ESHE significantly reduced MMP- mRNA expression by 51.4% and 48.3%, respectively, compared to activated cells. In contrast, simulated gastrointestinal digestion only slightly altered the effect on MMP-9 mRNA expression: significant inhibition was only observed for 200 µg/mL RSHE (by 49.4%) and RESHE (by 41.5%) (Figure 9). MMP-9 activity was reduced by the SHE, ESHE, RSHE and RESHE in a concentration-dependent manner when compared to the TNF-α-stimulated CCD841CoN; all tested agents inhibited MMP-9 activity more strongly at all used concentrations compared to budesonide. Additionally, it is worth emphasizing that RSHE and RESHE caused a greater reduction of MMP-9 activity compared to the same concentrations of SHE and ESHE. At higher tested concentration (200 µg/mL), SHE, ESHE, RSHE and RESHE treatment resulted in MMP-9 activities of 55%, 61.5%, 45% and 42.5%, respectively (Figure 9).

### 3.7. Effect of the Tested Agents on VEGFR2 and HIF1α Expression

**Figure 10:**
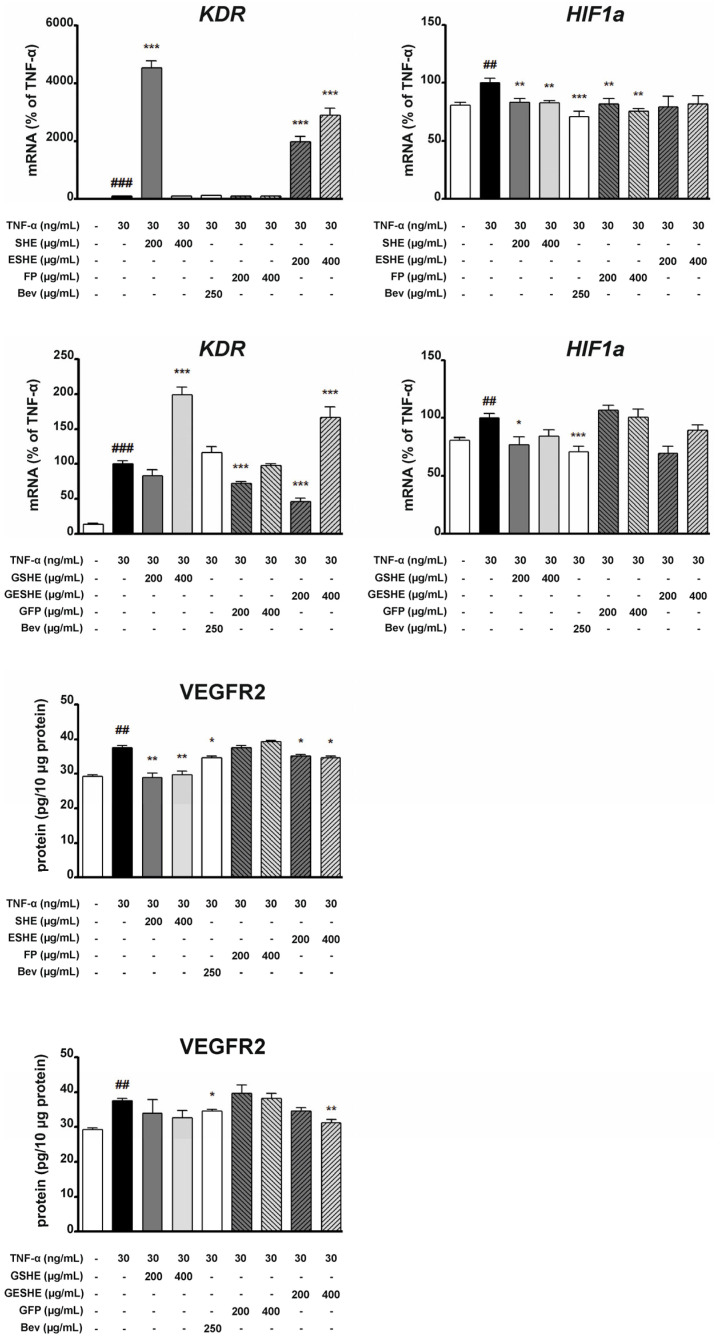
Effects of the tested agents on TNF-α-induced mRNA expression of KDR, HIF1α and TNF-α-induced protein expression of VEGFR2 in HIEC-6 cells. TNF-α-stimulated (30 ng/mL) HIEC-6 were treated with the tested agents (200, 400 µg/mL). The mRNA levels of KDR and HIF1α were measured by quantitative polymerase chain reaction (Q-PCR) after 16 h treatment. Protein expression of VEGFR2 in TNF-α-stimulated HIEC-6 cells was examined by an enzyme-linked immunosorbent assay after 24 h treatment. Budesonide (Bud, 1 µM) was used for comparison. Each value on the graphs represents the mean value ± SEM, n = 3 independent experiments. Significance of differences between means: ^##^ *p* < 0.01, ^###^ *p* < 0.001 versus control cells, * *p* < 0.05, ** *p* < 0.01, *** *p* < 0.001 versus TNF-α-stimulated cells.

In HIEC-6 cells, treatment with the non-digested agents (SHE, ESHE and FP) did not reduce the mRNA expression of KDR, i.e., gene encoding VEGFR2. In contrast, treatment with 200 µg/mL of GESHE and GFP reduced KDR mRNA expression by 54.3% and 27.7% compared to the TNF-α-stimulated HIEC-6 cells. The significant downregulation of VEGFR2 protein expression was detected at both concentrations for SHE and ESHE, as well as at higher concentration of GESHE. Interestingly, SHE demonstrated a stronger inhibitory effect compared to budesonide. In turn, SHE inhibited HIF-1α gene expression to 83.3% (200 µg/mL) and 82.8% (400 µg/mL) compared TNF-α-stimulated cells. Also, FP lowered HIF-1α expression in a concentration-dependent manner. Among the digested agents, only 200 µg/mL GSHE caused significant inhibition (Figure 10).

**Figure 11:**
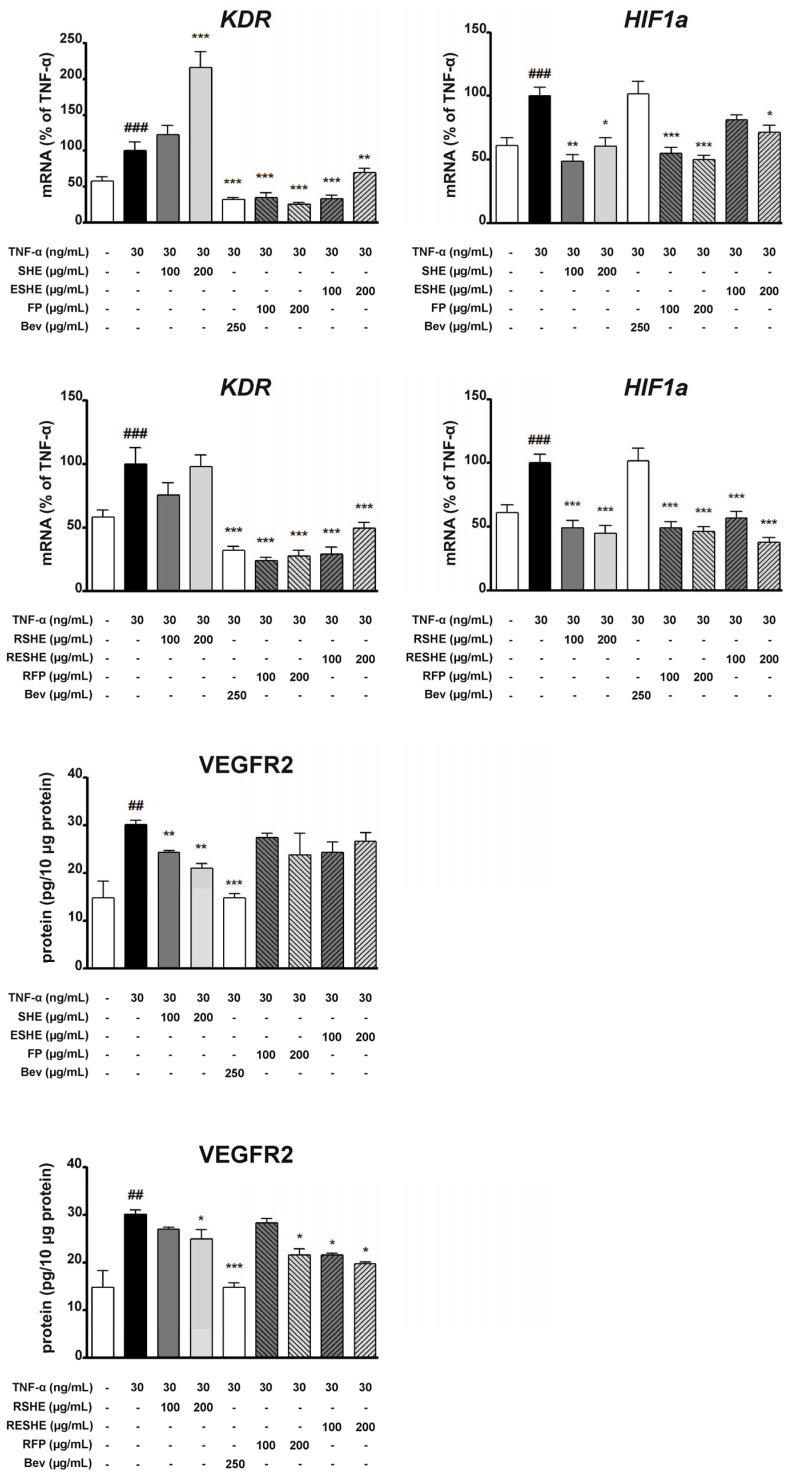
Effects of the tested agents on TNF-α-induced mRNA expression of KDR, HIF1α and TNF-α-induced protein expression of VEGFR2 in CCD841CoN cells. TNF-α-stimulated (30 ng/mL) CCD841CoN were treated with the tested agents (100, 200 µg/mL). The mRNA levels of KDR and HIF1α were measured by quantitative polymerase chain reaction (Q-PCR) after 16-h treatment. Protein expression of VEGFR2 in TNF-α-stimulated CCD841CoN cells was examined by an enzyme-linked immunosorbent assay after 24 h treatment. Budesonide (Bud, 1 µM) was used for comparison. Each value on the graphs represents the mean value ± SEM, n = 3 independent experiments. Significance of differences between means: ^##^ *p* < 0.01, ^###^ *p* < 0.001 versus control cells, * *p* < 0.05, ** *p* < 0.01, *** *p* < 0.001 versus TNF-α-stimulated cells.

In CCD841CoN cells, FP and ESHE, and their digested forms (RFP and RESHE) the significantly inhibited KDR mRNA expression. For all concentrations, RFP demonstrated stronger inhibition than budesonide, which caused a decrease by 67.7%. In contrast, VEGFR2 protein expression was downregulated by only one non-digested agent, SHE; VEGFR2 protein level decreased to 24.3 (100 µg/mL) and 21.1 pg/10 μg protein (200 µg/mL) compared to TNF-α-stimulated CCD841CoN cells that were not exposed to SHE (30.2 pg/10 μg protein). Regarding the digested agents, RSHE and RFP inhibited VEGFR2 protein expression at 200 µg/mL, and RESHE at 100 µg/mL (by 8.6/10 μg protein) and 200 µg/mL (by 10.4/10 μg protein) in a concentration-dependent manner (Figure 11).

The tested agents significantly lowered the mRNA expression of HIF-1α. All digested agents inhibited the mRNA expression in a concentration-dependent manner. At 200 µg/mL, RSHE, RESHE and RFP reduced mRNA expression by 55.3%, 62.5% and 53.7% compared to the TNF-α-activated cells. In addition, SHE and FP (both concentrations), and 200 µg/mL ESHE significantly reduced HIF-1α mRNA expression. Incubation with 200 µg/mL SHE, FP and ESHE resulted in 39.7%, 50% and 28.2% decreases in HIF-1α mRNA expression relative to the TNF-α-triggered cells, respectively (Figure 11).

### 3.8. Effect of the Tested Agents on the Activity of the Nuclear Factor Kappa B (NF-κB) Signalling Pathway

As NF-κB is an important transcription factor complex controlling the expression of mediators associated with inflammation, the next stage assessed the impact of the tested agents on the mRNA and protein expression of NF-κB. In HIEC-6 cells, all examined non-digested agents significantly inhibited the mRNA expression of RELA. Interestingly, SHE caused greater inhibition than Bay 11-7082 for both tested concentrations, resulting in RELA mRNA expression being 49.8% (200 µg/mL) and 62.4% (400 µg/mL) of the TNF-α-induced cells not exposed to any tested agents. In turn, ESHE decreased the mRNA expression in a concentration-dependent manner by 17.7% (200 µg/mL) and 26.6% (400 µg/mL).

**Figure 12:**
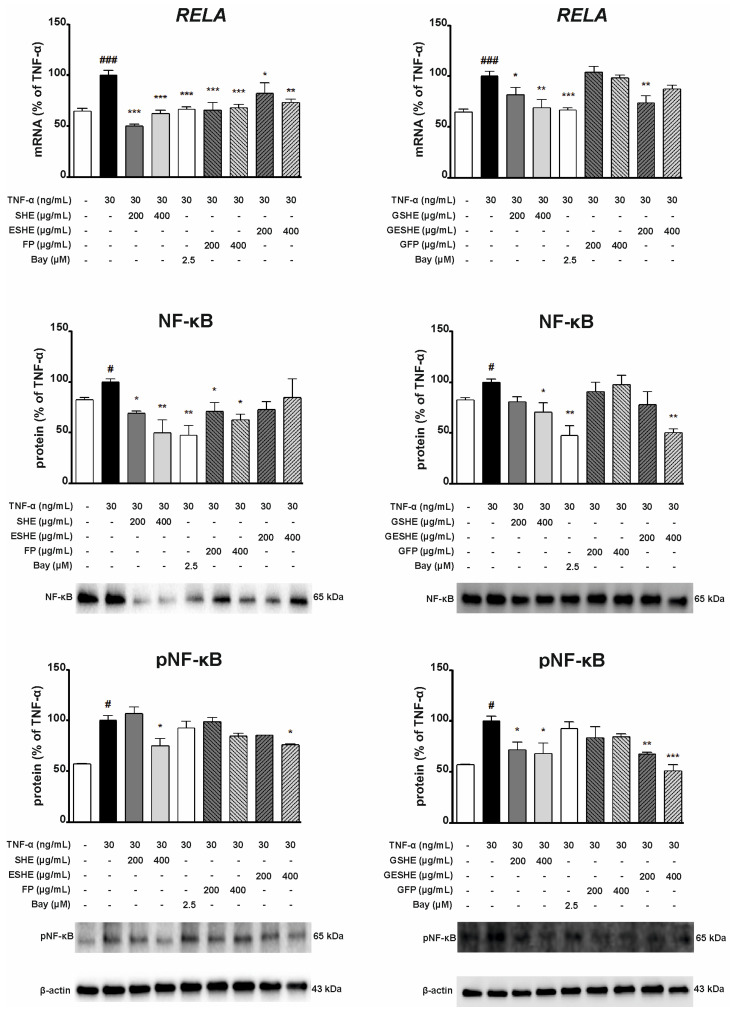
Effects of the tested agents on the NF-κB pathway induced by TNF-α in HIEC-6 cells. The mRNA expression of RELA was measured by quantitative polymerase chain reaction (Q-PCR) in TNF-α-stimulated (30 ng/mL) HIEC-6 cells after treatment with the tested agents for 16 h. Protein expression of NF-κB and p-NF-κB in TNF-α-stimulated (30 ng/mL) HIEC-6 cells, following six-hour incubation with various concentrations of the tested agents (200, 400 µg/mL), was examined by Western blot assay. Bay 11-7082 (2.5 µM) was used a positive control. The quantification of protein bands was performed after densitometric analysis scanning of immunoblots and normalized to β-actin level. The normalized amount of protein in TNF-α-stimulated cells was taken as 100%. Each value on the graphs represents the mean value ± SEM, n = 3 independent experiments. Significance of differences between means: ^#^ *p* < 0.05, ^###^ *p* < 0.001 versus control cells, * *p* < 0.05, ** *p* < 0.01, *** *p* < 0.001 versus TNF-α-stimulated cells.

For the digested agents, significant changes were reported for GSHE and 200 µg/mL of GESHE. In HIEC-6 cells, GSHE reduced RELA mRNA expression by 18.3% (200 µg/mL) and 31.3% (400 µg/mL) compared to TNF-α-stimulated HIEC-6 cells. Both concentrations of SHE and FP reduced NF-κB protein expression, as did 400 µg/mL of GSHE and GESHE. However, the greatest inhibition was observed for SHE, resulting in reduction by 30.8% (200 µg/mL) and 50.2% (400 µg/mL). Also, 400 µg/mL of SHE and ESHE inhibited p-NF-κB protein expression; in addition, GSHE and GESHE treatment caused 28.4% and 32.6% decreases at 200 µg/mL, and 31.8% and 49.2% decreases at 400 µg/mL, respectively (Figure 12). GSHE and GESHE are able to inhibit the NF-κB pathway by both reducing NF-κB protein expression and downregulating pathway activation.

In CCD841CoN cells, RELA gene expression was significantly downregulated by all tested agents. SHE, ESHE and FP reduced the mRNA expression by 39%, 29.2% and 33.2%, at 100 µg/mL, respectively, and by 41.6%, 31.5% and 47.7% at 200 µg/mL, respectively. Incubation with SHE, RSHE and RESHE decreased NF-κB protein expression. SHE treatment reduced NF-κB protein expression to 67.5% (100 µg/mL) and 63.3% (200 µg/mL) of the TNF-α-stimulated values, RESHE suppressed protein expression of NF-κB by 38.1% at 100 µg/mL and 33% at 200 µg/mL. Interestingly, 200 µg/mL of SHE and both concentrations of RESHE reduced NF-κB protein expression more strongly than the used Bay inhibitor.

**Figure 13:**
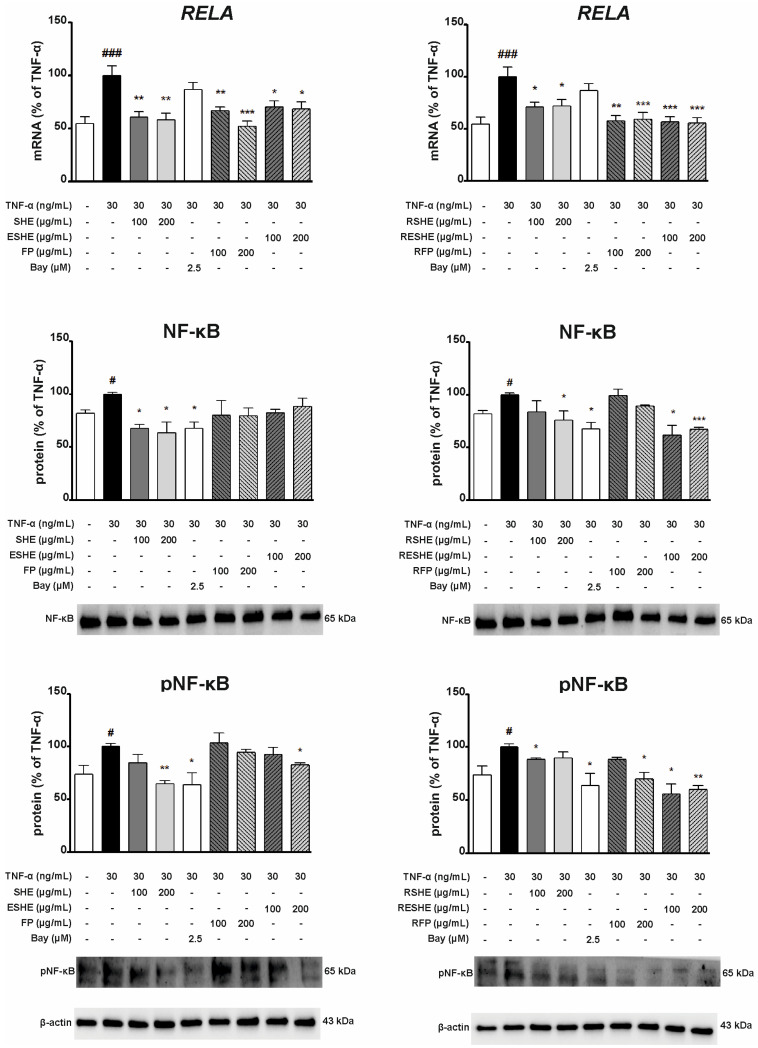
Effects of the tested agents on the NF-κB pathway induced by TNF-α in CCD841CoN cells. The mRNA expression of RELA was measured by quantitative polymerase chain reaction (Q-PCR) in TNF-α-stimulated (30 ng/mL) CCD841CoN cells after treatment with the tested agents for 16 h. Protein expression of NF-κB and p-NF-κB in TNF-α-stimulated (30 ng/mL) CCD841CoN cells, following six-hour incubation with various concentrations of the tested agents (100, 200 µg/mL), was examined by Western blot assay. Bay 11-7082 (2.5 µM) was used a positive control. The quantification of protein bands was performed after densitometric analysis scanning of immunoblots and normalized to β-actin level. The normalized amount of protein in TNF-α-stimulated cells was taken as 100%. Each value on the graphs represents the mean value ± SEM, n = 3 independent experiments. Significance of differences between means: ^#^ *p* < 0.05, ^###^ *p* < 0.001 versus control cells, * *p* < 0.05, ** *p* < 0.01, *** *p* < 0.001 versus TNF-α-stimulated cells.

The treatments also inhibited p-NF-κB expression. RESHE downregulated p-NF-κB protein expression in the TNF-α-induced CCD841CoN cells by 44.4% (100 µg/mL) and 40.1% (200 µg/mL); both cases demonstrated stronger inhibition than the Bay inhibitor. Moreover, a slight decline in p-NF-κB protein expression was noted after treatment with 200 µg/mL of SHE, ESHE and RFP, as well as 100 µg/mL of RSHE, demonstrating that these agents are able to inhibit NF-κB pathway activation (Figure 13).

### 3.9. Effect of the Tested Agents on the Activity of the Extracellular Signal-Regulated Kinase (ERK) Signalling Pathway

**Figure 14:**
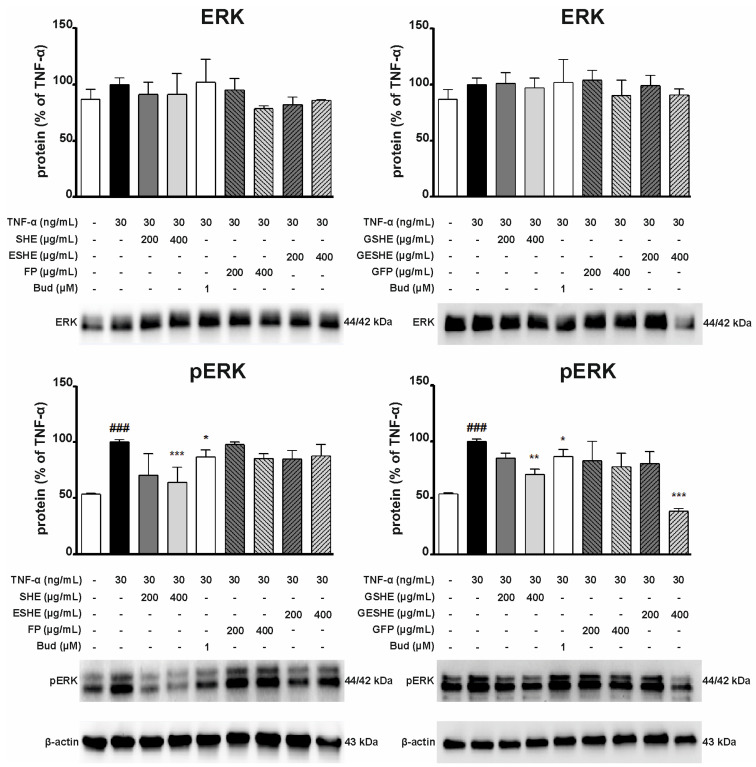
Effect of the tested agents on the phosphorylation of ERK signalling pathway in TNF-α-stimulated HIEC-6 cells. TNF-α-stimulated (30 ng/mL) HIEC-6 cells were treated with SHE (200 and 400 µg/mL) for six hours. The protein expression was examined by Western blot assay. Budesonide (Bud, 1 µM) was used a positive control. The quantification of protein bands was performed after densitometric analysis scanning of immunoblots and normalized to β-actin level. The normalized amount of protein in TNF-α-stimulated cells was taken as 100%. Each value on the graphs represents the mean value ± SEM, n = 3 independent experiments. Significance of differences between means: ^###^ *p* < 0.001 versus control cells, * *p* < 0.05, ** *p* < 0.01, *** *p* < 0.001 versus TNF-α-stimulated cells.

**Figure 15:**
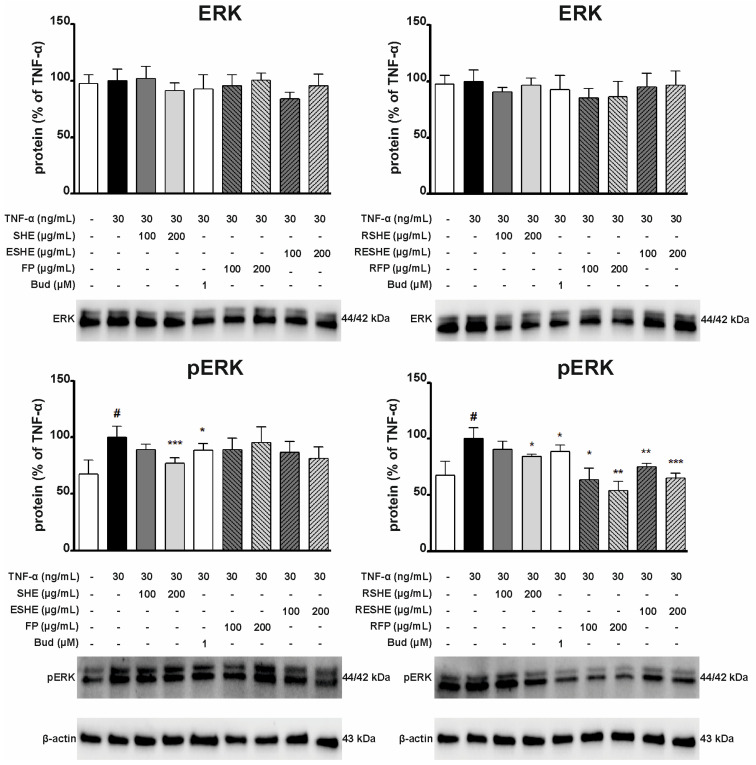
Effect of the tested agents on the phosphorylation of ERK signalling pathway in TNF-α-stimulated CCD841CoN cells. TNF-α-stimulated (30 ng/mL) CCD841CoN cells were treated with SHE (100 and 200 µg/mL) for six hours. The protein expression was examined by Western blot assay. Budesonide (Bud, 1 µM) was used a positive control. The quantification of protein bands was performed after densitometric analysis scanning of immunoblots and normalized to β-actin level. The normalized amount of protein in TNF-α-stimulated cells was taken as 100%. Each value on the graphs represents the mean value ± SEM, n = 3 independent experiments. Significance of differences between means: ^#^ *p* < 0.05, versus control cells, * *p* < 0.05, ** *p* < 0.01, *** *p* < 0.001 versus TNF-α-stimulated cells.

As the ERK pathway takes part in the progression of IBD, the next stage examined the impact of tested agents on pathway activity following induction by TNF-α, which strongly stimulates phosphorylation in the pathway. In HIEC-6 cells, SHE, GSHE and GESHE significantly reduced phosphorylated ERK (p-ERK) protein expression. After six-hour incubation with 400 µg/mL of SHE, GSHE and GESHE, p-ERK levels were reduced by 35.8%, 29.3%, and 61.8%, respectively. Interestingly, these reductions were greater than for budesonide (Figure 14). In turn, in CCD841CoN cells, treatment with 200 µg/mL of SHE and RSHE significantly reduced p-ERK protein expression by 22.8% and 15.8%. Additionally, p-ERK protein expression was reduced by 24.6% (100 µg/mL) and 34.8% (200 µg/mL) after incubation with RESHE, and fell to 63.4% (100 µg/mL) and 54.2% (200 µg/mL) of TNF-α-activated CCD841CoN cells values after treatment with RFP (Figure 15). These results indicate that these agents inhibited pathway activation by reducing its phosphorylation state.

### 3.10. Effect of the Tested Agents on the Activity of the Protein Kinase B (Akt) Signalling Pathway

**Figure 16:**
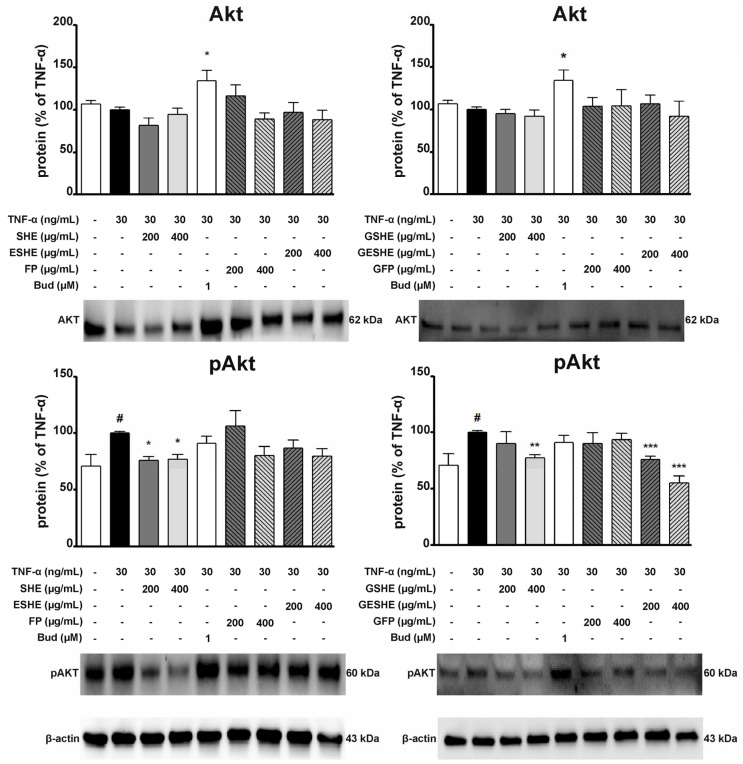
Effect of the tested agents on the phosphorylation of Akt signalling pathway in TNF-α-stimulated HIEC-6 cells. TNF-α-stimulated (30 ng/mL) HIEC-6 cells were treated with SHE (200 and 400 µg/mL) for six hours. The protein expression was examined by Western blot assay. Budesonide (Bud, 1 µM) was used a positive control. The quantification of protein bands was performed after densitometric analysis scanning of immunoblots and normalized to β-actin level. The normalized amount of protein in TNF-α-stimulated cells was taken as 100%. Each value on the graphs represents the mean value ± SEM, n = 3 independent experiments. Significance of differences between means: ^#^ *p* < 0.05, versus control cells, * *p* < 0.05, ** *p* < 0.01, *** *p* < 0.001 versus TNF-α-stimulated cells.

**Figure 17:**
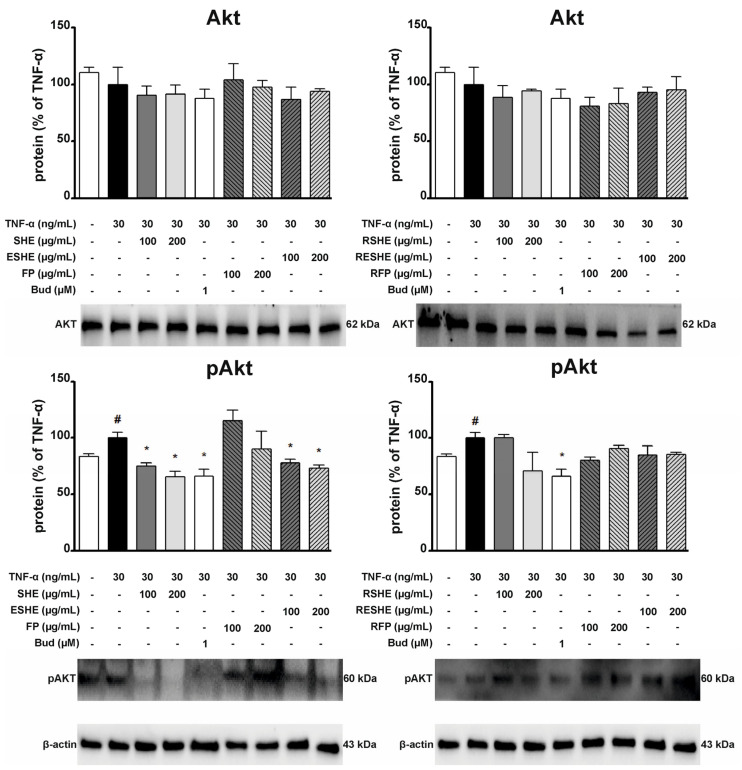
Effect of the tested agents on the phosphorylation of Akt signalling pathway in TNF-α-stimulated CCD841CoN cells. TNF-α-stimulated (30 ng/mL) CCD841CoN cells were treated with SHE (100 and 200 µg/mL) for six hours. The protein expression was examined by Western blot assay. Budesonide (Bud, 1 µM) was used a positive control. The quantification of protein bands was performed after densitometric analysis scanning of immunoblots and normalized to β-actin level. The normalized amount of protein in TNF-α-stimulated cells was taken as 100%. Each value on the graphs represents the mean value ± SEM, n = 3 independent experiments. Significance of differences between means: ^#^ *p* < 0.05, versus control cells, * *p* < 0.05, versus TNF-α-stimulated cells.

Due to the fact that Akt is involved in the inflammatory response in IBD [22], we also examined the impact of the tested agents on TNF-α-induced activation of the Akt signalling pathway. In HIEC-6 cells, treatment with SHE, GSHE, and GESHE significantly reduced the protein expression levels of phosphorylated Akt (p-Akt). After six hours of incubation with 400 µg/mL of SHE, GSHE, and GESHE, p-Akt levels decreased by 22.8%, 22.4%, and 39.9%, respectively (Figure 16). In contrast, in CCD841CoN cells, treatment with 100 µg/mL of SHE and ESHE significantly decreased p-Akt protein expression by 24.7% and 22.1%, respectively. Additionally, p-Akt expression was reduced to 65.8% and 73.0% of TNF-α-stimulated CCD841CoN cell levels following treatment with 200 µg/mL of SHE and ESHE, respectively. However, the digested forms of the tested agents did not inhibit Akt phosphorylation, indicating a lack of protective effects of encapsulation in preserving phenolic compound activity against Akt activation in CCD841CoN cells (Figure 17).

## 4. Discussion

The treatment of IBD remains a challenge, and there is considerable interest in identifying new therapeutic options and adjunctive agents. The present study evaluated the efficacy of SHE (*Humulus lupulus* L.), its encapsulate and their digested forms in in vitro model of IBD.

The study examined the effects of SHE treatment on CCD841CoN cells derived from large intestine epithelium, the site of UC, and HIEC-6 cells derived from small intestine epithelium, the site of CD. The inflammatory response was stimulated using TNF-α, which play an important role in mediating intestinal and colonic inflammation in IBD. It activates signalling pathways associated with cell differentiation and death, and stimulates the expression of pro-inflammatory mediators, particularly interleukins. In addition, IBD is characterized by increased expression of TNF-α, which is associated with disruption of the mucosal barrier and remodelling of the extracellular matrix [23]. Interestingly, TNF-α is able to induce the expression of type IV collagenases (MMP-2, MMP-9), pro-angiogenic factors (VEGF, VEGFR2) and HIF-1α, which promote carcinogenesis in the colon, resulting in a worse patients’ prognosis [24,25].

As such, IBD treatments should contain compounds that can inhibit the inflammatory response and oxidative stress in the bowels, and bestow anti-cancer properties, preventing against IBD-related CRC. Moreover, plant-based nutrients are receiving growing attention as prophylactic and therapeutic agents due to their natural origin, potential low toxicity and low costs of production compared to synthetic agents. Our findings indicate that SHE derived from *Humulus lupulus* could be a safe, pro-health treatment options. SHE contains a high TPC, as well as strong anti-oxidant potential.

The two main subclasses of phenolic compounds contained in SHE were the flavonols, represented mainly by quercetin derivatives, and hydroxycinnamic acids, represented mainly by chlorogenic acid and neochlorogenic acid. Flavonols have been found mitigate intestinal inflammation mainly by limiting the production of pro-inflammatory mediators, including TNF-α and nitric oxide (NO), and modulating gut microbiota [26,27,28]. The hydroxycinnamic acids are believed to inhibit the formation of colonic ulcers, stimulate the expression of antioxidant pathways and reduce the expression of pro-inflammatory cytokines [29,30]. Our results indicate that SHE inhibited the expression of pro-inflammatory cytokines, such as IL-1β, IL-6 and TNF-α, in both cell lines. It is important to emphasize that the study assessed the expression of TNF-α by the cultures, despite using the same cytokine to stimulate the cells; this was performed as TNF-α induces TNF-α expression in a positive feedback loop [31]. These cytokines are responsible for initiating and exacerbating the inflammatory process in the small intestine and colon, and for stimulating the release of other pro-inflammatory mediators, such eicosanoids or proteolytic enzymes, leading to damage of bowel tissue [32]. It is possible that SHE may suppress the inflammatory response by inhibiting these cytokines.

In turn, the type IV collagenases, represented herein by MMP-2 and MMP-9, are the main proteolytic enzymes capable of degrading extracellular matrix components, promoting mucosal damage, intestinal fibrosis and tissue remodelling [33]. These two are believed to play a significant role in promoting the progression of CRC [15]. Our results demonstrate that SHE inhibited MMP-2 and MMP-9 mRNA expression and activity, indicating that the extract can promote mucosal healing, prevent the development of complications observed in CD, such as stenosis or fistulas, and even exert a chemopreventive effect against IBD-related CRC. Furthermore, SHE downregulated the mRNA expression of HIF1a, a factor mediating angiogenesis and tumorigenesis, and the protein expression of VEGFR2, receptor associated with angiogenic processes [34], thus indicating that the tested extract has chemopreventive potential.

In the next stage of the study, we tried to identify the cellular mechanisms behind the observed anti-inflammatory and anti-cancer activities of SHE. In IBD, the production of cytokines and other pro-inflammatory mediators, the progression of UC and CD, disruption of the epithelial barrier and a higher histopathological score have been attributed to activation of the NF-κB signalling pathway [35]. In turn, activation of the ERK pathway in IBD is associated with the fibrotic processes of intestinal and colonic tissue, enhanced expression of inflammatory cytokines by epithelial cells and disease progression [36,37]. Our results indicate that SHE reduced the mRNA and protein expression of NF-κB in both cell lines. SHE treatment also lowered NF-κB signalling pathway activation, as noted by reduced expression of p-NF-κB. Also, SHE inhibited ERK pathway activation by limiting the ERK phosphorylation. Hence, SHE may exert its anti-inflammatory effect in IBD via suppression of NF-κB and ERK pathways. It is also worth emphasizing that the molecular mechanisms responsible for the inflammatory response in IBD are complex. The Akt signalling pathway plays an additional important role in the development and progression of IBD. It regulates apoptosis and proliferation of intestinal epithelial cells, as well as the maturation of dendritic cells involved in inflammation. Interestingly, Akt activity is associated with the regulation of various downstream target genes, including NF-κB. It has been shown that Akt phosphorylation leads to NF-κB activation, thereby promoting the progression of colitis [22,38,39]. In our study, we also demonstrated that SHE inhibited p-Akt protein expression, indicating that its anti-inflammatory effects may be mediated through multiple signalling pathways, including Akt.

In the next stage, the tested extract was digested in vitro to reflect human conditions. It was proven that digestive processes limit the stability, bioaccessibility and bioavailability of consumed phenolic compounds, decreasing their pro-health activity [40]. The extract was applied to HIEC-6 cells after incubation with gastric juice (GSHE) and to CCD841CoN cells after gastric and intestinal digestion (RSHE) to reflect human digestion. The composition analysis revealed a significant reduction in phenolic compound content, TPC and TF in the GSHE and RSHE extracts compared to SHE; lower levels were also noted in RSHE compared to GSHE. It was found that GSHE had weaker effects on HIEC-6 cells for all assessed molecules, except for p-NF-κB protein expression; this confirms that the amount and activity of the polyphenols contained in SHE were lowered by digestion. In contrast, no such change was noted in CCD841CoN cells after treatment with RSHE. The mRNA expression of MMP-9 and RELA, as well as the protein expression of TNF-α, VEGFR2, NF-κB, p-NF-κB and p-ERK were decreased after RSHE treatment lesser than after incubation with non-digested SHE.

To enhance the stability and the efficacy of action and improve the bioavailibity of phenolic compounds in organism after oral administration, mainly its solubility, short residence time and low permeability in the gut, the polyphenol-rich extract should be administered as a highly stabilized delivery system [41]. One way of maintaining sufficient biological activity in labile materials, such as polyphenols, by encapsulating them [42].

In the food industry, microencapsulation is used to protect a core bioactive material from degradation by reducing its reactivity, adjusting the location and time of its release, optimizing its physical properties to enable easier handling, and maintaining the active molecular form until the time of consumption [43]. A polyphenol-rich extract can be introduced into microcapsules by spray drying, which is characterized by relatively low cost and high availability of equipment [44,45]. It must be emphasized that such encapsulation appears to be an effective delivery system for pro-health agents, and the payloads are characterized by high bioavailability and bioaccessibility [46]. One particularly effective group of encapsulation materials comprises water soluble flaxseed polysaccharides (FP) extracted from *Linum usitatissimum* L. seeds. Flaxseeds are characterized by many nutritional properties [47]. The FP are obtained from flaxseed extruded mucilage when heated with water consisting of neutral and acidic fractions. It was showed that the flaxseeds extruded mucilage has hypolipidemic, anti-ulcer, anti-diarrheal, anti-diabetic and anti-cancer properties. In details, L-arabinose, D-xylose and D-galactose are the main constituent of the neutral fraction, while the acidic fraction contains L-rhamnose, L-fucose, L-galactose and D-galacturonic acid. Also, arabinoxylan with (1→4)-*β*-D-xylan backbone is a significant portion of a neutral fraction. The galactose and arabinose are attached either at 2 or 3 or both positions. In turn, the galactose and fucose are as branches at the backbone of (1→2)-linked α-L-rhamnopyranosyl and (1→4)-linked D-galactopyranosyluronic acid in an acidic fraction [48]. FP can be used to create delivery systems, are able to enhance activity of polyphenols and can act as independent ingredients [49]. Also, FP have antioxidant and anti-inflammatory activities, prebiotic potential and can be fermented in the large intestine to short chain fatty acids (SCFAs) with anti-inflammatory properties [50]. As such, in addition to improving the bioavailability of SHE, encapsulation with FP would also restore the disturbed gut dysbiosis observed in IBD, thus enhancing the anti-inflammatory effect. Therefore, encapsulation with FP was used to enhance the delivery of SHE, with the treatment being named ESHE. It is worth emphasizing that despite the lack of digestion, ESHE may induce an anti-inflammatory effect due to its high levels of phenolic compounds. Our results indicate that ESHE is able to exert anti-inflammatory effects, mainly by downregulating IL-1β and TNF-α mRNA and protein expression, and by inhibiting MMP-2, MMP-9, KDR and RELA mRNA expression, VEGFR2 and p-NF-κB protein expression, and MMP-9 activity in HIEC-6 cells; hence ESHE may prevent against tissue remodelling and angiogenesis in the small intestine in cases of CD.

In turn, ESHE decreased IL-1β, IL-6, TNF-α, MMP-2, MMP-9, KDR, HIF1a and RELA mRNA expression, IL-1β protein expression and MMP-2 and MMP-9 activity in CCD841CoN cells, demonstrating that it may mitigate inflammation in the colon observed in UC and CD, and may exert a chemopreventive effect against the development of CRC.

The simulated digestion of ESHE was performed to assess its impact on the pro-health activity of the encapsulated SHE. Our findings indicate that encapsulation prevented the loss of anti-inflammatory activity demonstrated by non-encapsulated SHE. In addition, GESHE and RESHE strongly inhibited the activation of NF-κB and ERK pathways. The observed differences in activity between the tested agents may result from their different compositions. For example, despite containing higher amounts of phenolic compounds than GESHE and RESHE, ESHE had significantly lower levels of flavan-3-ols but higher levels of prenylflavonoids. Moreover, GSHE was characterized by a higher total phenolic content despite lower levels of fla-van-3-ols compared to GESHE. However, it is worth emphasizing that the inhibition of pro-inflammatory cytokine expression by some tested agents was non-linear and not strictly concentration-dependent, particularly in the case of IL-6 mRNA expression in HIEC-6 cells following GSHE and GFP treatment, and IL-1β mRNA and protein expression in CCD841CoN cells after RFP treatment. These inconsistencies may result from the partial loss of phenolic compounds during the digestion process. Compared to the non-digested agents (SHE, FP), the digested agents (GSHE, GFP, RFP) contain lower amounts of phenolic compounds, which may reduce their inhibitory effects on IL-1β and IL-6 expression. In addition, GFP and RFP lack flavonols, which are known to exert strong anti-inflammatory activity by suppressing pro-inflammatory cytokines [51,52,53,54]. With this in mind, along with the fact that phenolics act in additive, synergistic, or antagonistic manners, it is possible that the anti-inflammatory potential of SHE may depend on the fact it contains phenolics belonging to various groups. Furthermore, the encapsulation process may prevent the loss of biological pro-health activities of SHE. In addition, the FP also has anti-inflammatory potential and so may complement the biological and synergistic activities of compounds included in SHE, thus influencing their anti-inflammatory potential.

## 5. Conclusions

This study is not without limitations. The applied in vitro model oversimplifies the pathophysiology of IBD, particularly CD. CD can affect the entire gastrointestinal tract, not just the small or large intestine, and the inflammation is transmural, unlike the mucosal inflammation typical of UC. Our cellular model does not reflect the complex inflammatory environment of IBD, which involves immune cells, fibroblasts, and an intact epithelial barrier. In addition, inflammation was artificially stimulated using only one pro-inflammatory cytokine. The results from the current study should be confirmed in vivo. Also, the applied concentrations of SHE are difficult to achieve in the digestive tract in relation to physiologically achievable levels after oral administration. However, encapsulation may improve the stability, bioaccessibility, and bioavailability of SHE, increasing the likelihood of achieving therapeutic concentrations in vivo.

In summary, this is the first study demonstrating that SHE may have a protective effect against IBD. This therapeutic effect is predominantly associated with inhibition of NF-κB, ERK, and Akt activity and the inhibition of pro-inflammatory cytokine expression in HIEC-6 and CCD841CoN cells. Moreover, SHE may exhibit chemopreventive potential against the development of inflammation-associated CRC. Furthermore, the tested encapsulated formulation of SHE appears to exert greater bioaccessibility than SHE alone. Importantly, the encapsulated formulation (ESHE) demonstrated improved bioaccessibility and maintained activity following simulated digestion, suggesting its promise as a delivery system.

Although our findings are preliminary and limited to cell-based models, they provide a theoretical basis for further investigation of SHE and its encapsulated form as potential adjuncts in IBD management. Future in vivo studies are warranted to evaluate their efficacy and safety in physiological conditions.

## Figures and Tables

**Table 1:** The content of phenolic compounds in individual agents.

Agent	Phenolic Compounds Amount [mg/100 g]	Hydroxycinnamic Acids Amount [mg/100 g]	Hydroxybenzoic Acids Amount [mg/100 g]	Flavan-3-Ols Amount [mg/100 g]	Flavonols Amount [mg/100 g]	Prenylflavonoids Amount [mg/100 g]
SHE	879.73 ± 3.21	207.16 ± 1.02	19.48 ± 0.20	9.53 ± 0.10	554.75 ± 4.21	88.81 ± 1.21
GSHE	305.11 ± 2.01	72.08 ± 0.68	13.49 ± 0.45	1.89 ± 0.04	209.03 ± 1.98	8.62 ± 0.25
RSHE	131.17 ± 0.95	27.30 ± 0.45	3.64 ± 0.10	nd	74.88 ± 0.85	25.35 ± 1.12
ESHE	543.03 ± 1.04	142.25 ± 1.02	12.77 ± 0.21	1.80 ± 0.08	347.32 ± 2.11	38.89 ± 0.89
GESHE	192.53 ± 0.98	48.25 ± 0.60	9.96 ± 0.11	4.87 ± 0.16	121.82 ± 1.01	7.63 ± 0.27
RESHE	230.76 ± 1.10	36.72 ± 1.11	6.07 ± 0.10	4.50 ± 0.10	151.04 ± 1.11	32.43 ± 0.79
FP	15.23 ± 0.38	8.77 ± 0.11	3.65 ± 0.43	nd	2.81 ± 0.30	nd
GFP	5.26 ± 0.23	4.46 ± 0.28	0.80 ± 0.10	nd	nd	nd
RFP	1.82 ± 0.08	1.82 ± 0.08	nd	nd	nd	nd

**Table 2:** The content of flavonols in individual agents.

Agent	Flavonols Amount [mg/100 g]									
	Quercetin	Quercetin 3-O-Glucoside	Quercetin 3-O-Arabinoside	Quercetin 3-O-Galactoside	Quercetin 3-O-Rutinoside	Quercetin 3-O-Rhamnoside	Kaempferol 3-O-Galactoside	Kaempferol 3-O-Rutinoside	Kaempferol 3-O-Glucoside	Total
SHE	5.91 ± 0.09	166.81 ± 1.00	63.30 ± 0.67	4.80 ± 0.05	148.39 ± 0.98	9.08 ± 0.90	54.88 ± 0.45	64.33 ± 0.67	37.25 ± 0.25	554.75 ± 4.21
GSHE	nd	58.25 ± 0.48	27.49 ± 0.29	0.66 ± 0.01	58.64 ± 0.30	4.49 ± 0.50	21.20 ± 0.30	21.85 ± 0.30	16.45 ± 0.10	209.03 ± 1.98
RSHE	nd	23.16 ± 0.20	7.37 ± 0.08	0.58 ± 0.02	21.15 ± 0.45	1.26 ± 0.10	8.19 ± 0.10	9.32 ± 0.10	3.85 ± 0.03	74.88 ± 0.85
ESHE	3.20 ± 0.08	100.17 ± 1.02	42.20 ± 0.45	4.70 ± 0.05	93.51 ± 0.98	6.13 ± 0.10	34.11 ± 0.25	38.30 ± 0.40	25.00 ± 0.30	347.32 ± 2.11
GESHE	nd	33.21 ± 0.34	16.18 ± 0.20	1.55 ± 0.01	34.79 ± 0.42	2.74 ± 0.34	11.90 ± 0.20	11.81 ± 0.23	9.64 ± 0.09	121.82 ± 1.01
RESHE	nd	43.15 ± 0.45	18.31 ± 0.21	2.27 ± 0.02	42.59 ± 0.51	2.77 ± 0.21	15.60 ± 0.12	16.92 ± 0.15	9.43 ± 0.10	151.04 ± 1.11
FP	nd	nd	nd	nd	nd	nd	0.91 ± 0.04	1.90 ± 0.01	nd	2.81 ± 0.30
GFP	nd	nd	nd	nd	nd	nd	nd	nd	nd	0
RFP	nd	nd	nd	nd	nd	nd	nd	nd	nd	0

**Table 3:** The content of hydroxycinnamic acids in individual agents.

Agent	Hydroxycinnamic Acids Amount [mg/100 g]					
	Caffeic Acid	Ferulic Acid	p-Coumaric Acid	Chlorogenic Acid	Neochlorogenic Acid	Total
SHE	20.64 ± 0.21	5.05 ± 0.20	14.33 ± 0.70	38.31 ± 0.87	128.83 ± 1.30	207.16 ± 1.02
GSHE	8.34 ± 0.10	7.55 ± 0.26	4.58 ± 0.20	11.00 ± 0.70	40.61 ± 0.98	72.08 ± 0.68
RSHE	3.29 ± 0.15	2.25 ± 0.10	10.64 ± 0.60	2.39 ± 0.11	8.73 ± 0.32	27.30 ± 0.45
ESHE	9.08 ± 0.25	4.53 ± 0.21	10.99 ± 0.50	25.81 ± 0.22	91.84 ± 1.07	142.25 ± 1.02
GESHE	5.85 ± 0.40	1.93 ± 0.09	9.34 ± 0.36	6.80 ± 0.30	24.33 ± 0.78	48.25 ± 0.60
RESHE	6.66 ± 0.41	1.90 ± 0.08	5.77 ± 0.20	5.00 ± 0.45	17.39 ± 1.01	36.72 ± 1.11
FP	nd	4.85 ± 0.11	3.92 ± 0.10	nd	nd	8.77 ± 0.11
GFP	nd	2.43 ± 0.10	2.03 ± 0.30	nd	nd	4.46 ± 0.28
RFP	nd	0.98 ± 0.06	0.84 ± 0.05	nd	nd	1.82 ± 0.08

**Table 4:** The content of hydroxybenzoic acids in individual agents.

Agent	Hydroxybenzoic Acids Amount [mg/100 g]				
	Vanillic Acid	Syringic Acid	3,4-Dihydroxybenzoic Acid	4-Hydroxybenzoic Acid	Total
SHE	nd	4.41 ± 0.20	9.06 ± 0.09	6.01 ± 0.10	19.48 ± 0.20
GSHE	3.44 ± 0.18	1.65 ± 0.10	7.10 ± 0.35	1.30 ± 0.10	13.49 ± 0.45
RSHE	0.72 ± 0.01	0.53 ± 0.08	2.30 ± 0.15	0.09 ± 0.02	3.64 ± 0.10
ESHE	nd	2.65 ± 0.10	5.52 ± 0.19	4.60 ± 0.5	12.77 ± 0.21
GESHE	2.55 ± 0.11	1.00 ± 0.09	5.94 ± 0.10	0.47 ± 0.01	9.96 ± 0.11
RESHE	1.85 ± 0.09	1.07 ± 0.11	3.04 ± 0.14	0.11 ± 0.03	6.07 ± 0.10
FP	nd	0.52 ± 0.08	nd	3.13 ± 0.10	3.65 ± 0.43
GFP	nd	nd	nd	0.80 ± 0.06	0.80 ± 0.06
RFP	nd	nd	nd	nd	nd

**Table 5:** The content of prenylflavonoids in individual agents.

Agent	Prenylflavonoids Amount [mg/100 g]			
	Isoxanthohumol	Xanthohumol	8-Prenylnaringenin	Total
SHE	6.25 ± 0.10	72.70 ± 1.11	9.86 ± 0.45	88.81 ± 1.21
GSHE	1.14 ± 0.10	7.04 ± 0.35	0.44 ± 0.04	8.62 ± 0.25
RSHE	3.09 ± 0.34	20.76 ± 1.01	1.50 ± 0.50	25.35 ± 1.12
ESHE	4.94 ± 0.36	32.01 ± 1.12	1.94 ± 0.10	38.89 ± 0.98
GESHE	2.18 ± 0.17	5.04 ± 0.30	0.41 ± 0.03	7.63 ± 0.27
RESHE	5.63 ± 0.46	24.93 ± 1.10	1.87 ± 0.40	32.43 ± 0.79
FP	nd	nd	nd	nd
GFP	nd	nd	nd	nd
RFP	nd	nd	nd	nd

**Table 6:** The antioxidant properties of the tested agents with general polyphenol content and flavonoid content. The general polyphenol content and flavonoid content were expressed as gallic acid equivalent (GAE) and quercetin equivalent (QE) per 100 g of individual agents, respectively.

Agent	Total Polyphenol Content [mg GAE/100 g]	Total Flavonoid Content [mg QE/100 g]	ABTS [mM TE/100 g]	DPPH [mM TE/100 g]	FRAP [mM Fe^2+^/100 g]
SHE	4418.48 ± 1.91	2295.71 ± 1.56	52.91 ± 1.34	34.07 ± 3.29	14.97 ± 0.98
GSHE	1304.44 ± 1.25	353.11 ± 0.98	13.04 ± 1.27	1.47 ± 0.22	9.26 ± 0.31
RSHE	775.01 ± 0.98	302.80 ± 1.03	11.92 ± 1.14	0.51 ± 0.09	5.19 ± 0.30
ESHE	2758.32 ± 1.98	1389.72 ± 1.65	29.51 ± 3.31	16.79 ± 3.18	7.64 ± 0.23
GESHE	1510.40 ± 1.45	601.33 ± 1.21	20.19 ± 3.01	5.91 ± 0.27	13.66 ± 0.40
RESHE	1741.70 ± 0.98	939.38 ± 1.45	36.16 ± 2.45	1.38 ± 0.21	12.67 ± 0.38
FP	242.49 ± 1.65	103.49 ± 1.45	5.53 ± 0.15	16.85 ± 3.08	2.90 ± 1.45
GFP	219.22 ± 0.78	62.84 ± 0.55	8.01 ± 0.20	3.82 ± 0.10	3.43 ± 0.20
RFP	337.61 ± 0.86	51.05 ± 0.61	13.65 ± 0.98	4.24 ± 0.09	4.06 ± 0.22

## Data Availability

Data are available from the corresponding author on reasonable request.

## Supplementary Materials

The following supporting information can be downloaded at: https://www.mdpi.com/article/10.3390/cells14141099/s1, Supplementary Table S1. The range of calibration, the coefficient of determination (R2), recovery, LOD, and LOQ of the investigated phenolic compounds and prenylflavonoids determined by UHPLC-DAD.

## Abbreviations

CD, Crohn’s disease; CRC, colorectal cancer; ESHE, encapsulate of spent hop extract; FP, flaxseed polysaccharides; IBD, inflammatory bowel diseases; MMP, matrix metalloproteinase; SHE, spent hop extract; TNF-α, tumour necrosis factor alpha; UC, ulcerative colitis.
