# Supplementary material for: Spent Hop (Humulus lupulus L.) Extract and Its Flaxseed Polysaccharide-Based Encapsulates Attenuate Inflammatory Bowel Diseases Through the Nuclear Factor-Kappa B, Extracellular Signal-Regulated Kinase, and Protein Kinase B Signalling Pathways
Source: Cells. 2025 Jul 17;14(14):1099. doi: 10.3390/cells14141099 (PMC12293961; doi:10.3390/cells14141099)
Supplement: Supplementary file 1 [file cells-14-01099-s001.zip › cells-3722902-supplementary.pdf]

Supplementary Table S1. The range of calibration, the coefficient of determination (R2), recovery, LOD, and LOQ of the investigated phenolic compounds and prenylflavonoids determined by UHPLC-DAD.

Standard	Linear range (mg/L)	R2	Recovery (%)	LOD (mg/L)	LOQ (mg/L)
Quercetin 3-*O*-rutinoside	0.1-50.0	0.997	95.7	0.02	0.08
Quercetin 3-O-glucoside	0.1-50.0	0.996	97.1	0.02	0.08
Kaempferol 3-O-glucoside	0.1-50.0	0.995	94.9	0.03	0.09
Quercetin	0.1-50.0	0.998	98.9	0.03	0.10
4-Hydroxybenzoic acid	0.5-100.0	0.995	89.5	0.04	0.10
3,4-dihydroxybenzoic acid	0.5-100.0	0.997	103.5	0.03	0.09
Vanilic acid	0.5-100.0	0.995	105.8	0.03	0.09
Syringic acid	0.5-100.0	0.996	98.6	0.03	0.09
Caffeic acid	0.1-50.0	0.998	95.7	0.04	0.12
Neochlorogenic acid	0.1-50.0	0.996	96.5	0.03	0.10
Chlorogenic acid	0.1-50.0	0.999	94.7	0.03	0.10
p-Coumaric acid	0.1-50.0	0.997	91.5	0.03	0.09
Ferulic acid	0.1-50.0	0.995	104.3	0.03	0.09
Isoxanthohumol	0.1-50.0	0.997	89.9	0.03	0.09
8-Prenylnaringenin	0.1-50.0	0.996	85.9	0.03	0.07

LOD - limit of detection, LOQ – limit of quantification
